# Orai1 Channels Are Essential for Amplification of Glutamate-Evoked Ca^2+^ Signals in Dendritic Spines to Regulate Working and Associative Memory

**DOI:** 10.1016/j.celrep.2020.108464

**Published:** 2020-12-01

**Authors:** Mohammad Mehdi Maneshi, Anna B. Toth, Toshiyuki Ishii, Kotaro Hori, Shogo Tsujikawa, Andrew K. Shum, Nisha Shrestha, Megumi Yamashita, Richard J. Miller, Jelena Radulovic, Geoffrey T. Swanson, Murali Prakriya

**Affiliations:** 1 Department of Pharmacology, Northwestern University Feinberg School of Medicine, Chicago, IL 60611, USA; 2 Department of Psychiatry, Northwestern University Feinberg School of Medicine, Chicago, IL 60611, USA; 3 Present address: Department of Physiology, Nippon Medical School, Tokyo, Japan; 4 Present address: Department of Anesthesiology, Osaka City University School of Medicine, Osaka City, Japan; 5 Lead Contact

## Abstract

Store-operated Orai1 calcium channels function as highly Ca^2+^-selective ion channels and are broadly expressed in many tissues including the central nervous system, but their contributions to cognitive processing are largely unknown. Here, we report that many measures of synaptic, cellular, and behavioral models of learning are markedly attenuated in mice lacking Orai1 in forebrain excitatory neurons. Results with focal glutamate uncaging in hippocampal neurons support an essential role of Orai1 channels in amplifying NMDA-receptor-induced dendritic Ca^2+^ transients that drive activity-dependent spine morphogenesis and long-term potentiation at Schaffer collateral-CA1 synapses. Consistent with these signaling roles, mice lacking Orai1 in pyramidal neurons (but not interneurons) exhibit striking deficits in working and associative memory tasks. These findings identify Orai1 channels as essential regulators of dendritic spine Ca^2+^ signaling, synaptic plasticity, and cognition.

## In Brief

Ca^2+^ signals in dendritic spines are critical for synaptic plasticity and cognitive functions. Maneshi et al. show that store-operated Orai1 channels are essential for amplifying neurotransmitter-evoked Ca^2+^ signals in dendritic spines of mouse hippocampal neurons. This function of Orai1 is crucial for long-term potentiation and hippocampal-dependent learning and memory.

## Graphical Abstract

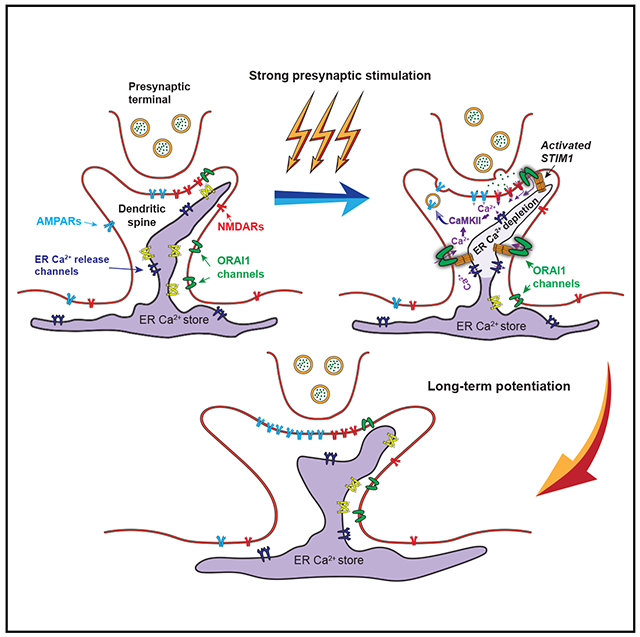

## INTRODUCTION

Ca^2+^ release-activated Ca^2+^ (CRAC) channels function as a major pathway for Ca^2+^ entry in many non-excitable cells where they mediate critical effector functions including gene expression, cytokine production, and cell migration (Prakriya and Lewis, 2015). Endowed with several unique biophysical features including very high Ca^2+^ selectivity and low unitary conductance, CRAC channels are typically activated in response to stimulation of G_q_-coupled receptors or receptor tyrosine kinases (Prakriya and Lewis, 2015). The ensuing depletion of endoplasmic reticulum (ER) Ca^2+^ stores activates the ER Ca^2+^ sensors, STIM1 and STIM2, which translocate to the ER-plasma membrane junctions to interact with and activate the channels formed by the Orai proteins in the plasma membrane, evoking a process known as store-operated Ca^2+^ entry (SOCE). Mammals express three Orai orthologs (Orai1-3), of which Orai1 is the best-studied member and is essential for SOCE in most non-excitable cells (Prakriya and Lewis, 2015). Human studies have revealed that loss-of-function Orai1 mutations cause severe immunodeficiencies and defects in muscle function frequently resulting in death in the first year of life, highlighting the vital importance of Orai1 channels for human health (Feske, 2019).

Recent evidence indicates that in addition to immune cells, Orai1 is expressed in many regions of the central nervous system (CNS) (Guzman et al., 2014; Lein et al., 2007), where it is implicated in a growing list of Ca^2+^-dependent cell functions (Kraft, 2015; Wegierski and Kuznicki, 2018). For example, in neural progenitor cells, astrocytes, and microglia, Ca^2+^influx through Orai1 regulates gene expression, gliotransmitter release, and the generation of inflammatory mediators (Deb et al., 2020; Gao et al., 2016; Heo et al., 2015; Somasundaram et al., 2014; Toth et al., 2019). Additionally, in spinal nociceptive neurons and hippocampal interneurons, Orai1 mediates SOCE and is implicated in the regulation of chronic pain and seizure-like activity (Dou et al., 2018; Hori et al., 2020; Majewski et al., 2019). By contrast, we know comparatively little about the role of Orai1 channels for Ca^2+^ signaling and effector functions in excitatory neurons. In this study, we address one aspect of this larger question: the contributions of Orai1 channels to synaptically evoked dendritic spine Ca^2+^ signals in hippocampal neurons and its implications for long-term potentiation (LTP).

In the archetypical form of LTP at the CA3-CA1 synapse in the hippocampus, Ca^2+^ elevations in dendritic spines of hippocampal CA1 neurons induce synaptic strengthening initially by increasing the number of AMPA-type ionotropic glutamate receptors (iGluRs) in postsynaptic densities and subsequently through gene expression and remodeling of spine structures (Herring and Nicoll, 2016). It is generally accepted that the iGluRs, especially NMDA receptors (NMDARs), have an obligate and privileged role in triggering plasticity at this synapse (Herring and Nicoll, 2016). NMDARs are thought to elicit Ca^2+^ elevations in dendritic spines both via their Ca^2+^ permeability and through their ability to trigger Ca^2+^ release from intracellular stores to drive essential molecular pathways, including Ca^2+^/calmodulin protein kinase II (CaMKII), that are implicated in mediating synaptic plasticity (Alford et al., 1993; Fitzjohnand Collingridge, 2002; Higley and Sabatini, 2012). However, how Orai channels fit into the prevailing model of dendritic spine Ca^2+^ signaling remains poorly understood and is only now beginning to be addressed.

Recent expression analysis and mRNA maps indicate that Orai1 is highly expressed in the hippocampus (Guzman et al., 2014). Moreover, knockdowns of Orai1 or STIM2 evoke impairments in spine maturation and morphogenesis in cultured neurons (Korkotian et al., 2017; Sun et al., 2014; Tshuva et al., 2017) while overexpression of Orai1 causes subtle alterations in learning in female mice (Maciąg et al., 2019), suggesting that Orai1 may directly regulate synaptic plasticity. However, answers to several fundamental questions remain unknown. What are dynamic properties of Orai1-dependent Ca^2+^ signals evoked by the neurotransmitter glutamate (Glu) in hippocampal neurons? How are they coupled to downstream signaling systems involved in LTP? And, importantly, what are the functional implications for cognitive functions? Surprisingly, even the basic question of whether Orai1 channels regulate synaptically evoked dendritic Ca^2+^ signals is unknown. In this study, we investigated these and related questions using cell-specific knockouts (KOs) of Orai1 and a variety of cellular, electrophysiological, and behavioral analysis. Our findings indicate that Orai1 channels play a critical role in dendritic signaling by promoting the amplification of NMDAR-mediated Ca^2+^ signals in dendritic spines, which is essential for inducing activity-dependent synaptic plasticity and the formation of working and associative memories.

## RESULTS

### Orai1 Mediates SOCE in Hippocampal Neurons

We previously showed that mice with a conditional deletion of Orai1 in the brain (generated by crossing Orai1^*fl/fl*^ mice with the nestin-Cre strain to yield Orai1^*fl/fl Nes-Cre*^ mice) show loss of SOCE in neural progenitor cells and astrocytes (Somasundaram et al., 2014; Toth et al., 2019). However, how deletion of Orai1 affects SOCE in mature excitatory neurons was not examined. To address this question, we began our studies by examining SOCE in primary cultured mouse hippocampal neurons. ER Ca^2+^ stores were depleted by the administration of the sarco/endoplasmic reticulum Ca^2+^ ATPase (SERCA) inhibitor, thapsigargin, and SOCE was determined from the rate of Ca^2+^ influx following re-addition of extracellular Ca^2+^ as previously described (Jairaman et al., 2015; Toth et al., 2019). These tests indicated that SOCE was strongly impaired in hippocampal neurons from Orai1^*fl/fl Nes-Cre*^ (Orai1 KO) mice (Figures S1A and S1B). Likewise, a more restricted deletion of Orai1 in excitatory neurons of the forebrain alone (Orai1^*fl/fl CaMKIIa-Cre*^ mice obtained by crossing Orai1^*fl/fl*^ mice with CamKIIa-Cre deleter strain; Tsien et al., 1996) also abolished SOCE in primary hippocampal neurons (Figures S1C and S1D), which in culture are known to be predominantly glutamatergic (Benson et al., 1994; Wu et al., 2015). These findings indicate that Orai1 is essential for mediating SOCE in excitatory hippocampal neurons, a role congruent with the established role of Orai1 in many immune, epithelial, and glial cells.

mRNA maps from the Allen Institute (Lein et al., 2007) suggest that Orai1 is highly expressed in the hippocampus, especially in the CA1 and CA3 pyramidal cell layers (Figure S1G). Consistent with this expression pattern, immunohistochemistry (IHC) with a monoclonal antibody (Guzman et al., 2014) revealed strong Orai1 expression in many regions of the hippocampus, including the CA1, CA3, and the dentate gyrus (Figure S1H). Orai1 labeling in CA1 was especially noticeable in the cell body and along the apical projections extending into the stratum radiatum. This pattern of Orai1 expression portends a number of potential roles for Orai1 channels in regulating Ca^2+^ signaling in hippocampal neurons, including those elicited by synaptic stimulation in dendrites, which we tested below.

### Deletion of Orai1 Attenuates Synaptically Evoked Ca^2+^ Signals in Dendritic Spines

**Figure 1.**
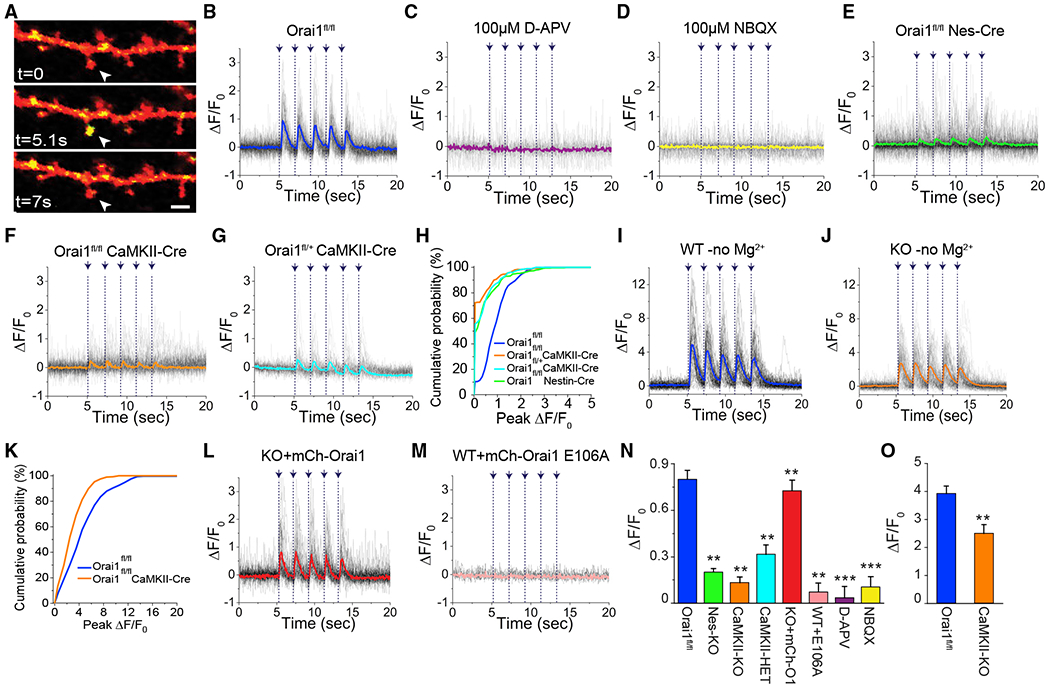
Deletion of Orai1 Impairs Synaptically Evoked Ca^2+^ Signals in Dendritic Spines (A) A section of a secondary apical dendrite of a hippocampal neuron expressing mCherry (red) and jGCaMP7f (yellow). MNI-Glu was uncaged at t = 5 s adjacent to the spine indicated by the arrowhead (4-ms pulse). Scale bar: 2 μm. (B) Dendritic spine jGCaMP7f fluorescence changes in response to Glu uncaging (4-ms pulses indicated by the arrows and vertical dotted lines) in neurons from WT (n = 8 cells, 67 spines) mice. The solid line shows the average of the individual spine Ca^2+^ responses (shown in lightly shaded lines). (C and D) The NMDAR and AMPAR antagonists, D-APV(100 μM) and NBQX(100 μM), respectively, block Glu-uncaging-evoked spine Ca^2+^ signals. (D-APV: n = 4 cells, 33 spines; NBQX: n = 5 cells, 42 spines). (E–G) Dendritic spine Ca^2+^ transients are strongly attenuated in Orai1 KO (Orai1^*fl/fl Nes-Cre*^) (n = 7 cells, 68 spines), Orai1^*fl/fl CaMKIIa-Cre*^ (n = 7 cells, 73 spines), and heterozygous (Orai1^*fl/+ CaMKIIa-Cre*^) mice (n = 9 cells, 108 spines). (H) Cumulative distribution of spine Ca^2+^ responses. Loss of Orai1 increases the fraction of failures (i.e., no Ca^2+^ response to stimulation). (I–K) In the absence of extracellular Mg^2+^, dendritic spine Ca^2+^ signals recover significantly in Orai1 KO (Orai1^*fl/fl CaMKIIa-Cre*^) neurons (WT, n = 6 cells, 61 spines; KO n = 9 cells, 98 spines). (L) Expression of WT Orai1 into Orai1 KO (Orai1^*fl/fl CaMKIIa-Cre*^) neurons restores dendritic spine Ca^2+^ signals (n = 5 cells, 43 spines). (M) Expression of the non-conducting E106D Orai1 pore mutant abrogates synaptically evoked spine Ca^2+^ signals in WT neurons (n = 9 cells, 97 spines). (N) Summary of the peak ΔF/F_0_ changes (mean ± SEM) in the indicated conditions (**p < 0.01, ***p < 0.001 from WT Orai1). (O) Summary of the peak ΔF/F_0_ changes (mean ± SEM) in the absence of extracellular Mg^2+^ (**p = 0.0038).

To interrogate the contribution of Orai1 channels in regulating Ca^2+^ signaling in dendritic spines, we employed the recently developed, high-performance genetically encoded Ca^2+^ indicator, jGCamP7f (*K_d_*~175 nM) (Dana et al., 2019). As the baseline fluorescence of jGCamP7f in the Ca^2+^-unbound state is low (Dana et al., 2019), we co-transfected cells with soluble mCherry to permit identification of spines (Figure 1A). Single dendritic spines with a diameter of ~1 μm and a head:neck ratio > 1.1, which are classically thought to represent mature spines containing the ER (Lai and Ip, 2013; Nimchinsky et al., 2002; Toresson and Grant, 2005; von Bohlen Und Halbach, 2009), were selected for spine Ca^2+^ imaging. To stimulate GluRs on these spines, we blocked action potentials with 1 mM tetrodotoxin (TTX) (to eliminate spontaneous signaling from other neurons) and uncaged 4-methoxy-7-nitroindolinyl (MNI)-Glu for 4 ms by focusing the galvanometer of the Nikon A1R confocal microscope at a single pixel located 0.5 μm from the center of the spine head (Figures 1A and S2D). With these stimulation conditions, we found that jGCaMP7f faithfully reported spine Ca^2+^ elevations not only with single Glu-uncaging pulses, but also with trains of low frequency (0.5 Hz) uncaging pulses (Figures S2D and S2E), indicating that the approach provides very good spatiotemporal resolution to monitor Ca^2+^ dynamics in dendritic spines. Progressively increasing the duration of the Glu-uncaging pulse enhanced the amplitude and duration of the spine Ca^2+^ rises, as expected with increasing concentration of free Glu (Figure S2F). We adjusted the length of the uncaging pulse to the lowest duration that reliably evoked [Ca^2+^]_i_ transients and found that a 4-ms uncaging pulse applied at a frequency of 0.5 Hz evoked [Ca^2+^]_i_ rises >90% of the time without affecting the activity of other nearby spines located 2 mm away (Figures S2D and S2E). This result is consistent with the compartmentalized nature of Ca^2+^ signaling within the restricted volume of dendritic spines (Sabatini et al., 2002).

It is well known that synaptically evoked Ca^2+^ signals in dendritic spines are triggered by NMDA GluRs (Sobczyk et al., 2005). In line with this evidence, the NMDAR blocker, D-APV (100 μM), abolished Glu-uncaging-evoked Ca^2+^ signals in wild-type (WT) neurons (Figure 1C). Likewise, as previously reported (Bloodgood et al., 2009; Nevian and Sakmann, 2004), NBQX, an antagonist of AMPA-type Glu receptors, attenuated the Glu-uncaging-evoked Ca^2+^ signals (Figure 1D). These results reaffirm the roles of NMDARs for triggering Ca^2+^ entry in dendritic spines and AMPA receptors (AMPARs) to evoke the requisite membrane depolarization to remove Mg^2+^ block from NMDARs and evoke spine Ca^2+^ elevations.

To assess the role of Orai1 channels for these spine Ca^2+^ signals, we compared Glu-uncaging-evoked Ca^2+^ responses in spines from WT and Orai1^*fl/fl Nes-Cre*^ mice (Figures 1B and 1E). These experiments revealed that Glu-uncaging-evoked Ca^2+^ signals were markedly impaired in neurons from Orai1^*fl/fl Nes-Cre*^ mice (Figures 1B, 1E, and 1N). Neurons from Orai1^*fl/fl CaMKIIa-Cre*^ mice also similarly showed striking attenuation of Glu-uncaging-evoked Ca^2+^ signals (Figures 1F and 1N). Fleterozygous Orai1^*fl/+ CaMKIIa-Cre*^ cells showed an intermediate phenotype (Figures 1G and 1N) indicating a gene dosage effect in the spine Ca^2+^ response. Analysis of the distribution of the Ca^2+^ responses following Glu-uncaging indicated that not only was the amplitude of the Ca^2+^ responses diminished in Orai1 KO neurons, but these cells also exhibited increased failures (Figure 1H). Together, these results reveal a critical role for Orai1 channels in magnifying spine Ca^2+^ signals following stimulation of GluRs.

Interestingly, removing extracellular Mg^2+^, which should remove Mg^2+^ block of NMDARs, significantly restored Ca^2+^ responses in Orai1 KO neurons (Figures 1I, 1J, and 1O) (84% ± 4.5% reduction in the presence of Mg^2+^ versus 36% ± 7.8% in absence). Thus, promoting the opening of NMDARs in Orai1 KO neurons moderately rescues dendritic spine Ca^2+^ signals. Moreover, in the absence of Mg^2+^, only a decrease in the amplitude of the Ca^2+^ response was observed in the Orai1 KO neurons, with no change in the failure rate (Figures 1K and 1O). Similarly, the magnitude of Ca^2+^ signal impairment in Orai1 KO neurons was diminished when the uncaging stimulation duration was increased from 4 to 64 ms (84% ± 4.5% inhibition at 4 ms versus 52% ± 6.2% at 64 ms) (Figure S2L). These results indicate that the contribution of Orai1 channels is most prominent at low stimulation strengths and decreases with stronger synaptic stimulation.

Several lines of evidence indicated that the attenuation of Glu-evoked spine Ca^2+^ signals in Orai1 KO neurons is directly related to the loss of Orai1 and not due to non-specific changes in spine function. First, re-introducing WT Orai1 expression in the Orai1 KO neurons by transfecting the neurons with WT Orai1 restored Glu-evoked spine Ca^2+^ signals to levels comparable to those seen in WT neurons (Figures 1L and 1N). Second, introduction of a dominant-negative Orai1 pore mutant, E106A Orai1, which abrogates Ca^2+^ permeation in Orai1 channels (Prakriya et al., 2006), abolished dendritic spine Ca^2+^ signals in WT neurons (Figures 1M and 1N). Third, acute pharmacological blockade of Orai1 channel activity by the trivalent Orai1 pore blocker, La^3+^, at a low concentration (1–2 μM) that is more selective for Orai1 over other cation channels (Jairaman and Prakriya, 2013), or the Orai1 inhibitor, BTP2 (Takezawa et al., 2006), significantly diminished the amplitude of the Ca^2+^ signals in WT neurons (Figures S2H and S2I). This result suggests that that the attenuated Ca^2+^ response in Orai1 KO neurons is not due to pleiotropic or developmental changes but requires Ca^2+^ entry across the plasma membrane. As seen in the Orai1 KO neurons, La^3+^ inhibition of spine Ca^2+^ signals was significantly reduced when extracellular Mg^2+^ was removed, indicating that the contribution of Orai1 channels is diminished with stronger levels of NMDAR stimulation (Figures S2J and S2K). Together, these results indicate that the Orai1 channel helps amplify GluR-evoked Ca^2+^ signals in dendritic spines, especially in physiologically relevant concentrations of extracellular Mg^2+^.

### Activation of Orai1-Mediated Ca^2+^ Influx Is Closely Linked to Depletion of ER Ca^2+^ Stores

**Figure 2.**
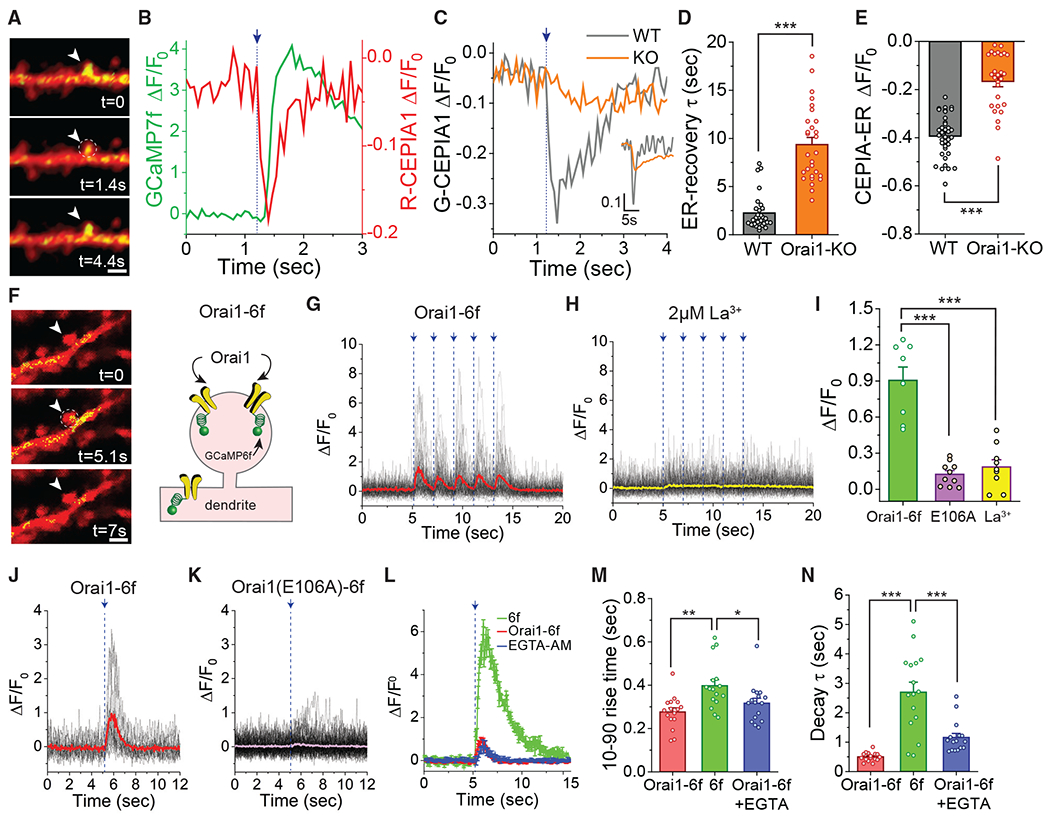
Glu Uncaging Evokes Rapid Ca^2+^ Store Release and Activation of Orai1 Channels (A) Images of a neuron expressing the ER marker, KDEL-mCherry (red), and G-CEPIA1er (green). The arrowhead shows the stimulated spine. Scale bar: 2 μm. (B) Time course of fluorescence changes in R-CEPIA1 er (red) and jGCaMP7f (green) in response to Glu uncaging (indicated by the arrow and vertical dotted line). (C) Representative traces of G-CEPIA1er in WT (Orai1^*fl/fl*^) and Orai1^*fl/fl CaMKIIa-Cre*^ (KO) in dendritic spines. (D) Recovery time constant (τ) of G-CEPIA1er in WT and Orai1 KO neurons (WT: n = 5 cells, 30 spines; Orai1-KO: n = 5, 30 spines, p < 0.001). (E) Summary of the ΔF/F_0_ change in WT and Orai1 KO neurons (WT: n = 6 cells, 37 spines, Orai1-KO: n = 5, 30, p < 0.001). (F) Images of Orai1-GCaMP6f (O1-6f) and mCherry co-expression in an Orai1^*fl/fl CaMKIIa-Cre*^ neuron. The cartoon on the right illustrates a schematic of Orai1-GCaMP6f. Scale bar: 2 μm. (G) Fluorescence changes of Orai1-GCaMP6f in the spine shown in (F). (H) A low dose of La^3+^ (2 μM) blocks the synaptically evoked dendritic spine Ca^2+^ signals. (I) Summary of the fluorescence changes in the indicated conditions (Orai1-GCaMP: n = 8 cells, 60 spines; Orai1 E106A-GCaMP6f: n = 10 cells, 95 spines; La^3+^:n = 9 cells, 85 spines, p < 0.001. All conditions compared to Orai1-GCaMP6f). (J and K) Representative examples of Orai1-GCaMP6f and E106AOrai1-GCaMP6f responses in an Orai1 KO neuron. (L) Traces showing the amplitude and time course of WT cells expressing GCaMP6f (6f) or Orai1 KO neurons expressing Orai1-GCaMP6f (O1–6f) in the absence (red traces) or presence (blue trace) of EGTA. EGTA-AM loading does not alter the Orai1-GCaMP6f signal or its time course. (M and N) Summary of the kinetics of Orai1-GCaMP6f signal in the absence (red) or presence (blue) of EGTA and in comparison to the soluble GCaMP6f signal (green) (n = 16 cells, 81 spines, p = 0.0013, p = 0.031, p < 0.001, p < 0.001 respectively). Summary data are represented as mean ± SEM.

As a store-operated channel, Orai1 activity should be closely linked to the filling state of ER Ca^2+^ stores (Luik et al., 2008). Therefore, to examine the relationship between Orai1-mediated Ca^2+^ influx and the dendritic spine ER Ca^2+^ store content, we monitored [Ca^2+^]_ER_ dynamics using the recently developed fluorescence resonance energy transfer (FRET)-based ER-targeted Ca^2+^ indicator, CEPIA1er (Figure 2A) (Suzuki et al., 2014, 2016). We found that each Glu-uncaging pulse evoked sharp and rapid decreases in [Ca^2+^]_ER_ in WT neurons (Figure 2B, red trace), which subsequently recovered fully with a time constant of ~2.2 ± 0.3 s (n = 6 cells, 30 spines) (Figures 2B, 2D, and S2M). Thus, synaptic stimulation evokes rapid depletion of ER Ca^2+^ stores, likely via calcium-induced Ca^2+^ release (CICR) as previously described (Alford et al., 1993; Emptage et al., 1999; Lee et al., 2016). Interestingly, simultaneous measurements of the cytosolic Ca^2+^ signal using jGCaMP7f indicated that in each spine, the cytosolic Ca^2+^ rise was closely coupled to, but slightly lagged, the decline in the CEPIA1erfluorescence (Figure 2B). On average, the latency between the fall in CEPIA1er fluorescence and the rise in the Orai1-mediated cytosolic Ca^2+^ signal was 92 ± 18 ms (n = 24 spines). These results argue that activation of the spine Ca^2+^ signal is closely linked to and follows depletion of ER Ca^2+^ stores, as expected for a store-operated channel.

By contrast, CEPIA1er measurements in Orai1 KO neurons showed several major differences. First, the magnitude of ER Ca^2+^ decrease was markedly diminished, with each Glu-uncaging pulse eliciting only a fraction of the decrement seen in WT neurons (Figures 2C and 2E). Second, recovery of [Ca^2+^]_ER_ following Glu uncaging was dramatically slowed (Figure 2D), with the [Ca^2+^]ER recovery time constant increasing 3-fold compared to WT neurons. Separately, administration of ionomycin to discharge intracellular stores indicated that Orai1 KO neurons have a significantly lower content of intracellular Ca^2+^ stores compared to WT neurons (Figure S1E). These findings show that loss of Orai1 dramatically impairs refilling of ER Ca^2+^ stores, resulting in lower levels of stored Ca^2+^ in Orai1 KO neurons and indicating that Orai1 helps maintain the filling state of ER Ca^2+^ stores.

### Synaptic Stimulation Evokes Rapid Activation of Orai1 in Dendritic Spines

We next sought to directly monitor the activity of Orai1 channels in dendritic spines during synaptic stimulation to better understand channel activation dynamics. Direct electrophysiological recordings of Orai1 currents in dendrites or dendritic spines are technically very difficult because the unitary conductance of Orai1 channels is extremely small (estimated to be only 25–100 fS for Ca^2+^) (Prakriya and Lewis, 2006, 2015), resulting in very small whole-cell Orai1 currents. However, recently, Cahalan and colleagues have shown that optical recordings of Orai1 channel activity offer an alternative to patch-clamp recording (Dynes et al., 2016, 2020). In this approach, a genetically encoded Ca^2+^ indicator (such as GCaMP6f) is fused to the intracellular C terminus of Orai1 to create a fusion protein of Orai1 with the Ca^2+^ indicator (Figure 2F). Orai1-GCaMP6f fusion is fully functional and retains normal electrophysiological properties including activation, inactivation, and Ca^2+^ selectivity (Dynes et al., 2016, 2020), providing a powerful approach to probe localized Orai1 channel activity. To obviate potential confounding issues of contributions from endogenous Orai1 channels, we expressed this construct in Orai1 KO (Orai1^*fl/fl CaMKIIa-Cre*^) hippocampal neurons together with mCherry to study Orai1 signals in mushroom-shaped spines.

These experiments revealed that Glu uncaging evokes a rapidly activating, transient Orai1-GCaMP6f fluorescence signal (Figure 2G). Orai1-GCaMP6f fluorescence rise was blocked by La^3+^ (2 μM) (Figures 2H and 2I) and abrogated by the non-conducting mutation, E106A Orai1 (Figures 2I–2K) (Prakriya et al., 2006). The 10%–90% rise time of Orai1-GCaMP6f fluorescence was 276 ± 18 ms (n = 16 cells, 81 spines) (Figure 2M), indicating that Orai1 channels turn on rapidly during synaptic stimulation and decayed with a tau of 500 ± 36 ms (n = 16 cells, 81 spines) (Figure 2N). A direct comparison of the amplitude of the ΔF/F_0_ signal between cells expressing the soluble, cytosolic GCaMP6f and the Orai1-GCaMP6f fusion protein showed that Orai1-GCaMP6f signal was considerably smaller but significantly faster in kinetics compared to soluble GCaMP6f (Figures 2L–2N). Importantly, loading cells with EGTA did not significantly affect the rise time of the Orai1-GCaMP6f signal (Figures 2L–2N), indicating that EGTA does not impair Orai1-GCaMP6f activation. These results are consistent with the notion that Orai1-GCaMP6f fluorescence changes provide a highly localized readout of Orai1 channel activity. The relatively rapid speed of Orai1 activation following Glu uncaging compared to the slower rates of Orai1 activation observed in non-excitable cells (Toth et al., 2019; Wu et al., 2006) suggests that Orai1 channels are positioned close to their gating ligand (likely STIM1 and/or STIM2) to permit rapid transfer of the channel gating signal over a time course relevant for evoking synaptic plasticity in dendritic spines.

### Loss of Orai1 Compromises AMPAR Insertion and Spine Morphogenesis

**Figure 3.**
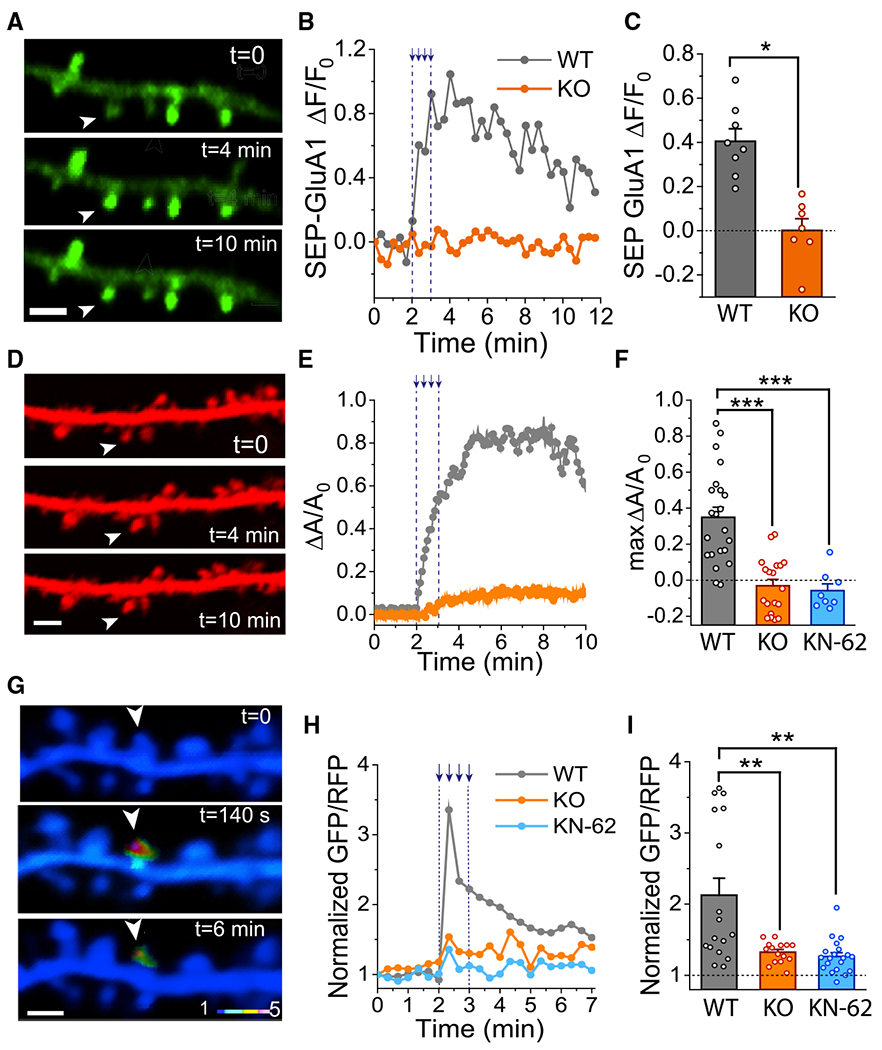
Deletion of Orai1 Impairs GluA1 Receptor Recycling, Spine Morphogenesis, and CaMKII Activation (A) Images showing SEP-GluA1 fluorescence in a hippocampal neuron co-transfected with mCherry (not shown) to permit easy identification of dendritic spines. The arrowhead shows the spine that was stimulated via Glu uncaging (four 64-ms uncaging pulses at 0.5 Hz). Scale bar: 2 μm. (B) Example traces showing changes in SEP-GluA1 fluorescence in single dendritic spines in response to Glu uncaging. (C) Summary of the SEP-GluA1 fluorescence changes in the indicated genotypes (WT: n = 8 cells, 36 spines; Orai1-KO: n = 7 cells, 30 spines, p = 0.01052). (D) Images (mCherry) showing enlargement of dendritic spines before and following Glu uncaging. The arrowhead denotes the stimulated spine. (E) Representative traces showing the relative changes in spine area following Glu uncaging. (F) Summary of the area (ΔA/A_0_) changes in spines from WT and Orai1 KO (Orai1^*fl/fl CaMKIIa-Cre*^) neurons (WT: n = 9 cells, 22 spines; Orai1-KO: n = 10 cells, 19 spines, p < 0.001; WT cells treated with 10μM KN62: n = 4 cells, 8 spines, p < 0.001). (G) Images showing the GFP/RFP fluorescence ratio of the CaMKII probe, Camui. (H) Changes in the GFP/RFP ratio of Camui in response to a tetanus of Glu uncaging. (I) Summary of the Camui GFP/RFP ratios in WT and Orai1 KO neurons (WT: n = 16 cells, 30 spines; Orai1 KO: n = 15 cells, 33 spines, p = 0.0045; WT cells treated with 10 μM KN62: n = 8 cells, 20 spines; p = 0.001634).

Dendritic Ca^2+^ signaling is closely linked to induction of synaptic plasticity (Bloodgood and Sabatini, 2007; Sala and Segal, 2014). In particular, elevations in spine Ca^2+^ signals are associated with insertion of new AMPARs into postsynaptic densities via exocytosis of receptor-containing vesicles, a process regulated by CaMKII (Lu et al., 2001; Malinow and Malenka, 2002). Thus, the strong impairment of Ca^2+^ signals in dendritic spines of Orai1 KO mice led us to consider whether cellular measures of synaptic plasticity might be affected in these mice. To test this hypothesis, we first imaged exocytosis of AMPARs using SuperEcliptic pHluorin (SEP)-tagged GluA1 receptors (Makino and Malinow, 2009). Following exocytosis of AMPAR-containing vesicles during LTP-like stimuli, fluorescence of SEP-GluA1 is dequenched, which is readily detected as an increase in GFP fluorescence. Cells were co-transfected with mCherry to enable measurements of spine volume following LTP induction (Figures 3A and 3D). This combination of measuring both SEP-GluA1 and mCherry fluorescence provides a powerful approach to simultaneously monitor both changes in GluA1 receptor recycling and spine size following synaptic stimulation (Makino and Li, 2013; Matsuzaki et al., 2004; Patterson et al., 2010).

We found that stimulating single spines with a train of four 64-ms Glu-uncaging pulses at 0.5 Hz evoked a several-fold increase in SEP fluorescence in the spine head (Figures 3A–3C) and robust increases in spine size (Figures 3D–3F). By contrast, in Orai1 KO neurons, Glu uncaging evoked a much smaller increase in SEP-GluA1 fluorescence (Figures 3B and 3C) or, in some cells, even a decrease in SEP-GluA1 fluorescence, possibly reflecting a long-term depression (LTD)-like process (Figure 3C). Moreover, examination of spine volume based on mCherry fluorescence revealed that spine enlargement was markedly reduced in the Orai1 KO neurons compared to controls (Figures 3E and 3F). These results indicate that loss of Orai1 in excitatory neurons strongly impairs spine enlargement and insertion of GluA1 receptors into the postsynaptic membrane in response to synaptic stimulation.

A large body of literature indicates that CaMKII is essential for transducing Ca^2+^ signals into changes in synaptic strength (Lisman et al., 2012). Therefore, using the FRET-based CaMKII probe, Camui (Erickson et al., 2011; Takao et al., 2005), we next examined if Orai1 channels regulate CaMKII activation in dendritic spines. These experiments revealed that Glu uncaging evoked robust increases in activation of Camui in many stimulated spines (Figures 3G and 3H). Activation of Camui in response to Glu uncaging was blocked by the CamKII inhibitor, KN-62 (Figure 3I), as was the spine enlargement in WT neurons (Figure 3F), reaffirming the central role of CaMKII in structural plasticity of dendritic spines (Matsuzaki et al., 2004). Notably, in Orai1 KO (Orai1^*fl/fl CaMKIIa-Cre*^) neurons, however, Glu uncaging failed to evoke Camui activation in all spines examined (Figures 3H and 3I). Thus, loss of Orai1 impairs CaMKII activation in dendritic spines of hippocampal neurons following synaptic stimulation.

### Loss of Orai1 Impairs Schaffer Collater-CA1 LTP

Given that our results show that deletion of Orai1 impairs synaptically evoked spine Ca^2+^ signaling, GluA1 recycling, spine enlargement, and CaMKII activation, we next considered the implications for LTP in brain slices. The archetypal form of LTP is the synaptic potentiation seen at the Schaffer collateral-CA1 synapse in the hippocampus, where intense synaptic activity evokes rhythmic bursts of Ca^2+^ signals in dendritic spines to strengthen synapses through insertion of new AMPARs and, in the longer term, via stable modification of dendritic spine structures (Higley and Sabatini, 2012).

**Figure 4.**
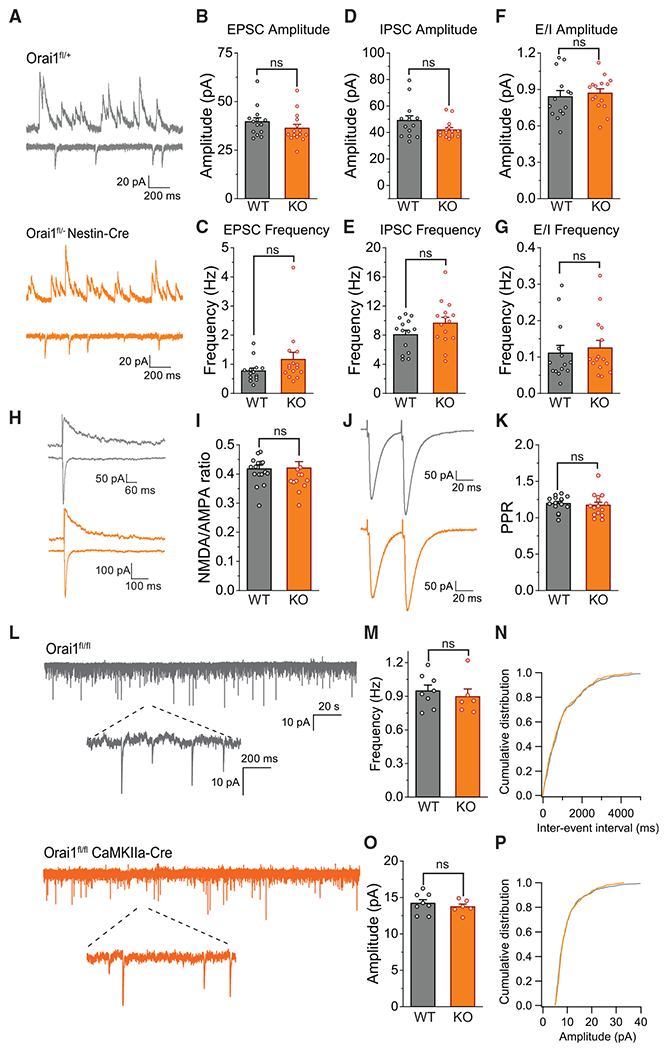
Basal Synaptic Transmission, Circuit Activity, and Presynaptic Function Are Not Affected by Orai1 Deletion (A) Representative recordings of spontaneous EPSCs and IPSCs recorded from a CA1 pyramidal neuron. Inward EPSC currents were recorded at a holding potential of −70 mV, and outward IPSC currents were obtained in the same neuron by switching the holding potential to 0 mM mV. (B and C) Amplitude (B) and frequency of EPSCs in WT (Orai1^*fl/fl*^) and Orai1 KO (Orai1^*fl/− Nes-Cre*^) slices (n = 14 WT, n = 15 Orai1-KO, p = 0.25, p = 0.09). (D and E) Amplitude (D) and frequency (E) of IPSCs in WT and Orai1 KO slices. (F) Ratio of the amplitudes of EPSCs and IPSCs in WT and Orai1 KO neurons (n = 14 WT, n = 15 Orai1-KO, p = 0.65). (G) EPSC/IPSC frequency ratio in the indicated genotype (n = 14 WT, n = 15 Orai1-KO, p = 0.64). (H) Representative traces showing the evoked AMPAR (inward) and NMDAR (outward) currents in a CA1 pyramidal cell. (I) NMDAR/AMPAR current ratios (n = 17 WT, n = 15 Orai1-KO, p = 0.907). (J and K) Paired EPSCs evoked by stimulation at a 50-ms interval. Paired-pulse ratio (PPR) was calculated as the ratio of the peak current of the second EPSC relative to that of the first EPSC (n = 15 WT, n = 15 Orai1-KO, p = 0.706). (L) mEPSCs in WT (Orai1^*fl/fl*^) and Orai1 KO (Orai1^*fl/fl CaMKIIa-Cre*^) slices in 1 μM TTX. (M–P) Comparison of the mean amplitude (p = 0.558) and frequencies (p = 0.478) of mEPSCs in WT (n = 8) and Orai1 KO mice (n = 6) and representative cumulative distributions of the interevent intervalsand amplitudes. ns, not significant.

As a first step, we investigated whether basal synaptic transmission in the CA1 hippocampus is affected in Orai1 KO mice, as this could directly impact the induction of LTP. Whole-cell patch clamp recording from CA1 pyramidal neurons in acutely dissociated brain slices showed that CA1 neurons received robust spontaneous excitatory and inhibitory synaptic activity in both WT and Orai1^*fl/fl Nes-Cre*^ mice (Figure 4A). The ratio of spontaneous excitatory/inhibitory input on CA1 neurons was comparable in WT and Orai1 KO mice both in amplitude and frequency (Figures 4B–4G), suggesting that overall baseline excitability of circuits within the CA1 was not grossly altered by the loss of Orai1. NMDA/AMPA current ratios measured at +40 and −70 mV were also not different between WT and Orai1 KO slices (Figures 4H and 4I), indicating that the relative numbers of NMDARs and AMPARs are similar in the two genotypes. Furthermore, neither the amplitude nor the frequency of mini excitatory postsynaptic currents (mEPSCs) in the presence of TTX was altered in Orai1 KO mice (Figures 4L–4P). These results suggest that basal measures of pre- and postsynaptic function in CA1 and the number of excitatory synapses in pyramidal neurons are unaffected by the absence of the Orai1 channel. Additionally, paired-pulse facilitation, a type of synaptic plasticity thought to have a presynaptic locus (Zucker and Regehr, 2002), was unaffected (Figures 4J and 4K), suggesting that short-term plasticity arising from presynaptic loci does not engage Orai1. Finally, analysis of spine size and density in Golgi-stained brain sections (Figures S3C–S3F) did not reveal differences between WT and Orai1 KO mice, indicating that loss of Orai1 does not elicit large-scale anatomical changes in hippocampal spines. Collectively, these results indicate that baseline hippocampal synaptic transmission is not regulated by Orai1 channels.

**Figure 5.**
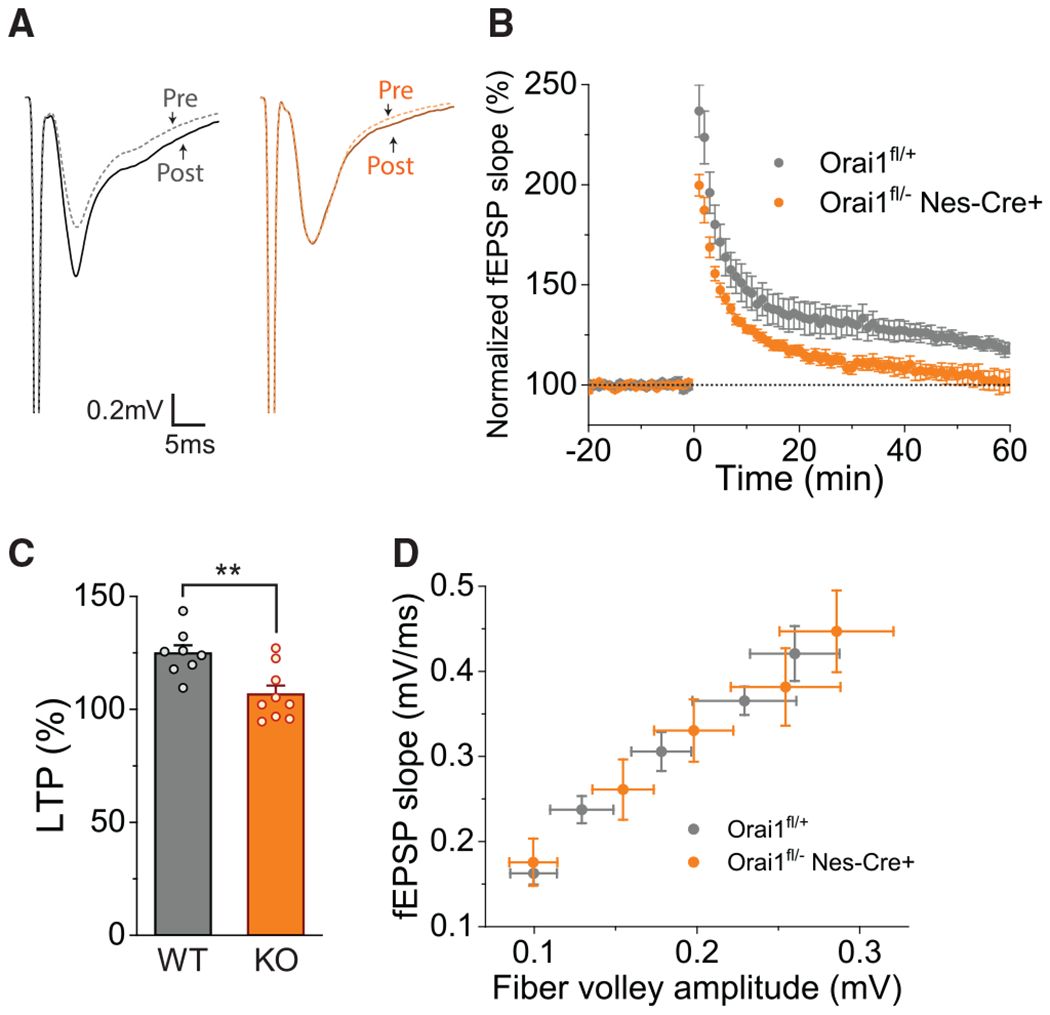
LTP Is Markedly Diminished in Orai1 KO Mice (A) Representative traces of the fEPSPs in WT (Orai1^*fl/fl*^) and Orai1 KO mice (Orai1^*fl/− Nes-Cre*^) before and after tetanus (1 × 100 Hz). (B) Slopes of the fEPSPs (normalized to the baseline values [which was set to 100%] during the pre-tetanus period) in WT and Orai1 KO mice. A 1 × 100 Hz tetanus was administered to the slices at t = 0 min. (C) Summary of the fEPSP slopes in WT (Orai1^*fl/fl*^) and Orai1 KO (Orai1^*fl/− Nes-Cre*^) mice at 60 min following tetanus. n = 8 slices from 4 WT mice, n = 9 slices from 4 Orai1 KO mice. p = 0.0041. (D) A plot of the fEPSP slope against increasing fiber-volley amplitude (input stimulus) in WT and Orai1 KO mice. Each point represents 8–12 recordings for WT slices and 10–12 recordings for the KO.

Strikingly, however, induction of CA1 LTP following a high-frequency tetanus (1 × 100 Hz) was markedly diminished in Orai1^*fl/fl Nes-Cre*^ mice (Figures 5A–5D). Specifically, recordings of the field excitatory postsynaptic potentials (fEPSPs) indicated that fEPSP slopes at 60 min following tetanus were not different from control values in Orai1 KO brain slices in both male and female mice, whereas in WT slices they were 125% of the control (Figures 5B and 5C). Notably, as excitatory/inhibitory current ratios were normal in Orai1 KO mice (Figures 4F and 4G) and GABA_A_ receptor antagonists were not present in the LTP experiments, the results rule out potential contributions due to changes in inhibitory circuits. Analysis of presynaptic fiber volley amplitude versus fEPSPs with increasing stimulus strength indicated that WT and Orai1 KO slices have comparable input-output relationships for postsynaptic responses (Figure 5D), ruling out again alterations in baseline synaptic transmission in CA1 as a contributing cause. A similarly lower degree of LTP was also seen following administration of the Orai1 channel inhibitor, BTP2, in WT slices (Figure S3A), suggesting that the decrease in LTP in the Orai1 KO mice is not due to developmental alterations in neuronal circuits. These results and the several lines of evidence presented in Figure 4 argue that the loss of CA1 LTP in the Orai1 KO mice is not due to generalized alterations in synaptic transmission or synapse function. Instead, the results are consistent with a functional deficit in Ca^2+^ signaling downstream of postsynaptic GluR activation that drives synaptic plasticity.

### Loss of Orai1 Compromises Learning and Memory

The findings indicating that Orai1 regulates dendritic Ca^2+^ signals and CA1 LTP led us to next examine potential implications for cognitive behaviors involving learning and memory. To address this question, we studied the Orai1^*fl/fl Nes-Cre*^ KO mice in a suite of learning and sensorimotor tests, including the Y-maze for short-term working memory and trace fear conditioning for associative memory, both of which involve the hippocampus (Gilmartin et al., 2014; Kraeuter et al., 2019). We also analyzed sensorimotor function using the Rotarod test to assess balance and coordination and open field to assess general locomotion and exploratory behavior (Gao et al., 2011).

**Figure 6.**
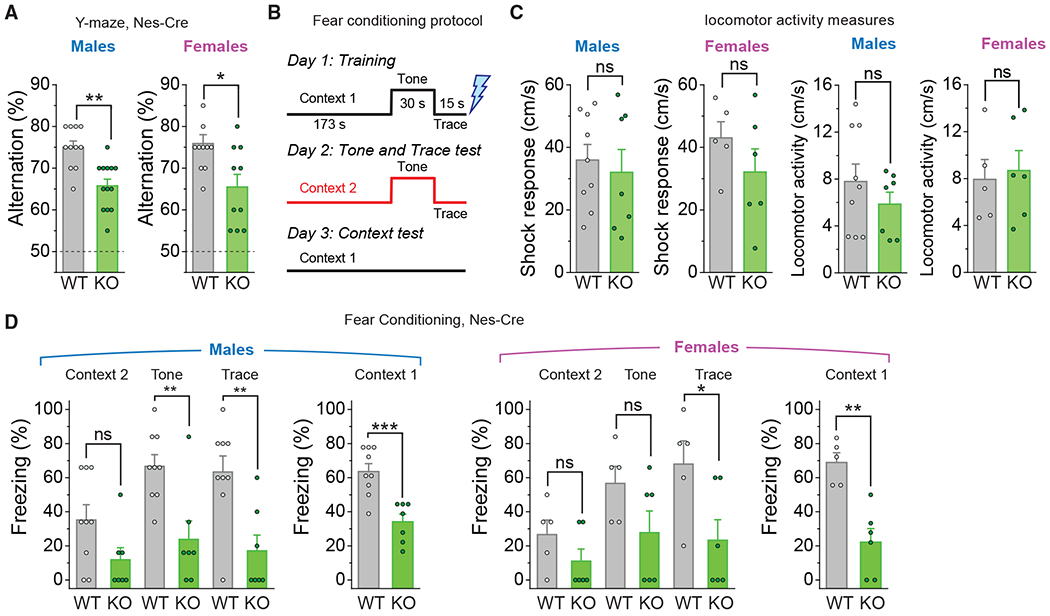
Orai1 KO Mice Are Impaired in Spatial Working and Associative Memory (A) Summary of spontaneous alternations in the Y-maze for male and female WT (Orai1^*fl/fl*^) and Orai1 KO (Orai1^*fl/fl Nes-Cre*^) mice. Male mice (n = 11 WT; n = 13 Orai1 KO, p = 0.0018), female mice (n = 11 WT; n = 10 Orai1 KO, p = 0.0109). Dashed line denotes chance (50% alternation). (B) Schematic of the fear-conditioning protocol. On day 1, mice in a conditioning chamber (context 1) were exposed to a conditioned stimulus (tone and trace interval), followed by an unconditioned stimulus (foot shock, in blue). On day 2, freezing response to the conditioned tone and trace (in a neutral context 2) was measured. On day 3, freezing response to the conditioned context 1 was measured. (C) Shock responses and average locomotor activity in the indicated genotypes on day 1. (D) Percentage of freezing to context 2, tone, trace, and context 1 in WT and Orai1 KO mice. Males: n = 9 WT, 7 KO. p values are as follows: context2, p = 0.0717; tone, p = 0.0035; trace, p = 0.0039; context1, p = 0.00051. Females: n = 5 WT, n = 6 KO. p values are as follows: context2, p = 0.1875; tone, p = 0.11725; trace, p = 0.03544; context1, p = 0.00134). ns, not significant.

These behavioral studies revealed that the Orai1 KO mice had significant deficits compared to WT littermate controls (Figure 6). In the Y-maze, male and female Orai1 KO mice showed a smaller percentage of spontaneous alternation compared to WT mice (Figure 6A). Likewise, in the fear-conditioning test, Orai1 KO mice had striking defects in both tone-dependent and contextual fear conditioning (Figure 6D). Our protocol included a 15-s trace interval between the tone (conditioned stimulus) and foot shock (unconditioned stimulus) to strengthen the hippocampal dependence of the association (Figure 6B) (Raybuck and Lattal, 2014). Loss of Orai1 did not affect locomotor activity in response to either the naive context or the electric shock (Figure 6C), ruling out differences in the sensation of the shock itself or potential alterations in locomotion. These results demonstrate that the loss of Orai1 compromises working memory and associative learning. Because these learning tasks rely on proper hippocampal function (Kandel, 2001), the results suggest that a fundamental aspect of hippocampal learning is impaired due to loss of Orai1 channels.

In contrast to compromised performance in the learning and memory tests, we found no deficits of balance, coordination, or general locomotion in the Orai1 KO (Orai1^*fl/fl Nes-Cre*^) mice using the Rotarod and open-field tests (Figures S4A and S4B). These results indicate that sensorimotor behaviors are not significantly affected by loss of Orai1 in the brain.

### Selective Loss of Orai1 in Excitatory Neurons Is Sufficient to Impair Learning and Memory

Cognitive functions related to learning and memory are closely tied to synaptic plasticity of excitatory synapses, especially in the hippocampus (Lynch, 2004). Given that our results showed a robust defect in Ca^2+^ signals and synaptic plasticity in dendritic spines of excitatory Orai1 KO neurons, we therefore hypothesized that mice lacking Orai1 in excitatory neurons alone might also show significant impairments in hippocampal-dependent learning and memory tasks. To address this question, we next examined behavioral learning in excitatory neuron-specific Orai1 KO (Orai1^*fl/fl CaMKIIa-Cre*^) mice. As a comparison, we also studied mice lacking Orai1 only in inhibitory neurons (Orai1^*fl/fl Gad2-Cre*^ mice) (Hori et al., 2020).

**Figure 7.**
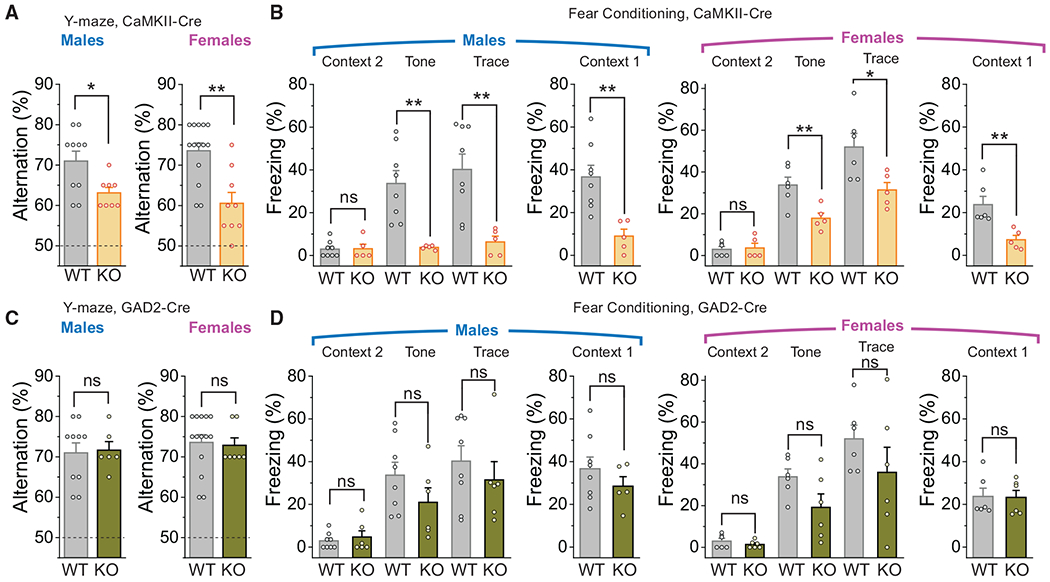
Selective Deletion of Orai1 in Excitatory Neurons Also Impairs Learning and Memory (A) Summary of the spontaneous alternations in the Y-maze for male and female WT (Orai1^*fl/fl*^) and Orai1 KO (Orai1^*fl/fl CaMKIIa-Cre*^) mice. Male mice: n = 10 WT; n = 8 Orai1 KO. p = 0.018. Female mice: n = 14 WT; n = 9 Orai1 KO. p = 0.00589. Dashed line denotes chance (50% alternation). (B) Summary of the freezing behaviors of WT (Orai1^*fl/fl*^) and Orai1 KO (Orai1^*fl/fl CaMKIIa-Cre*^) mice in the fear-conditioning protocol. Fear conditioning was quantified using Freezeframe software. Male mice: n = 8 WT; n = 5 Orai1 KO. p values are as follows: context2, p = 0.946; tone, p = 0.0028; trace, p = 0.0044; context1, p = 0.00331. Female mice: n = 6 WT; n = 5 Orai1 KO. p values are as follows: context2, p = 0.795; tone, p = 0.0081; trace, p = 0.0288; context1, p = 0.00647. (C) Y-maze in Gad2-Cre Orai1 KOs. Summary of the alternations in WT (Orai1^fl/fl^) and inhibitory neuron-specific Orai1 KOs (Orai1^*fl/fl Gad2-Cre*^) mice. Male mice: n = 10 WT; n = 6 Orai1 KO. p = 0.854. Female mice: n = 14 WT; n = 7 Orai1 KO, p = 0.816. (D) Summary of the freezing behavior in the fear-conditioning protocol in WT (Orai1^*fl/fl*^) and inhibitory neuron-specific (Orai1^*fl/fl Gad2-Cre*^) mice. Male mice: n = 8WT; n = 6 Orai1 KO. p values are as follows: context2, p = 0.551; tone, p = 0.1905; trace, p = 0.446; context1, p = 0.316. Female mice: n = 6WT; n = 6 Orai1 KO. p values are as follows: context2, p = 0.335; tone, p = 0.0775; trace, p = 0.2661; context1, p = 0.931.

These tests revealed that Orai1^*fl/fl CaMKIIa-Cre*^ mice have striking deficits in the learning and memory tasks (Figure 7). Specifically, WT mice showed 71% (males) and 72% (females) spontaneous alternation in the Y-maze, compared to 63% (males) and 60% (females) in the Orai1 KO mice (Figure 7A). This impairment is similar to the reduction in performance seen in the Orai1^*fl/fl Nes-Cre*^ mice (Figure 6A). Likewise, in the fear-conditioning test, both male and female Orai1^*fl/fl CaMKIIa-Cre*^ mice showed significant deficits in tone-dependent and contextual fear conditioning (Figure 7B). By contrast, Orai1^*fl/fl Gad2-Cre*^ mice performed at levels comparable to WT mice in the Y-maze (Figure 7C) and did not display significant differences in the fear conditioning test (Figure 7D). A direct comparison of the learning and memory behaviors in Orai1^*fl/fl Nes-Cre*^, Orai1^*fl/fl CaMKIIa-Cre*^, and Orai1^*fl/fl Gad2-Cre*^ mice (normalized to the respective WT values in each case) showed that Orai1^*fl/fl Nes-Cre*^ and Orai1^*fl/fl CaMKIIa-Cre*^ mice had significant deficits compared to WT and Orai1^*fl/fl Gad2-Cre*^ mice, both in the Y-maze and fear-conditioning tests (Figure S5). Taken together, these results indicate that Orai1 plays a major role in regulating cognitive functions involving learning and memory by controlling dendritic Ca^2+^ signaling and synaptic plasticity at excitatory synapses.

## DISCUSSION

Store-operated Orai1 channels have been extensively studied in non-excitable cells, especially immune cells where they function as highly Ca^2+^ -selective channels to regulate essential processes such as gene transcription, motility, and exocytosis (Prakriya and Lewis, 2015). Orai1 is also expressed widely in the nervous system (Guzman et al., 2014), yet the properties and physiological roles of Orai1 channels in the CNS, especially in forebrain neurons, are not well defined. In this study, we show that Orai1 channels are important regulators of Glu-evoked Ca^2+^ elevations in dendritic spines and LTP at Schaffer collateral-CA1 synapses. Ca^2+^ signals in dendritic spines of hippocampal neurons are strongly diminished by deletion of Orai1, by acute pharmacological blockade of Orai1 activity, and by the non-conducting Orai1 pore mutant, E106A. Moreover, loss of Orai1 leads to striking deficits in cognitive functions related to short-term and associative memory. In addition to revealing an essential Ca^2+^ entry mechanism for regulating synaptic plasticity and cognition, these results identify Orai1 channels as relevant molecular targets for the cognitive decline seen in neurodegenerative diseases and following brain injuries.

The idea that intracellular stores and store-operated Ca^2+^ channels contribute to synaptic plasticity originated from general observations nearly two decades ago showing that pharmacological store depletion protocols (thapsigargin, cyclopiazonic acid [CPA]) and non-specific inhibitors of Ca^2+^ signaling such as 2-APB and SKF36965 impair synaptically evoked Ca^2+^ transients in dendritic spines and LTP at CA3-CA1 synapses (Alford et al., 1993; Baba et al., 2003; Emptage et al., 1999, 2001). However, subsequent efforts to elucidate the underlying mechanisms stalled because the molecular components of SOCE were unknown. The discovery of Orai1 as the pore-forming subunit of the CRAC channel (Feske et al., 2006; Prakriya et al., 2006) initiated a renaissance in efforts to use molecular tools to more mechanistically probe the contributions of SOCE for neuronal Ca^2+^ signaling and downstream physiological functions. With this new information, Segal and colleagues found that RNAi-mediated Orai1 knockdown or dominant-negative mutants of Orai1 impaired spine maturation and spine enlargement following chemical LTP stimulation in rat neuronal cultures (Korkotian et al., 2017; Tshuva et al., 2017). Additionally, knockdown or inhibition of the ER Ca^2+^ sensor, STIM2, attenuated spine maturation in cultured hippocampal neurons (Sun et al., 2014; Zhang et al., 2015). Conversely, overexpression of STIM1 diminished LTD and improved contextual learning (Majewski et al., 2017). Together, these studies signaled growing recognition for the involvement of SOCE in regulating synaptic plasticity (Moccia et al., 2015; Wegierski and Kuznicki, 2018). Yet, the mechanistic links between Orai1 channel activity and dendritic Ca^2+^ signaling, hippocampal LTP, and cognitive behaviors have remained poorly understood.

The results presented in this work address these fundamental questions. Our findings indicate that loss or blockade of Orai1 channels profoundly impairs of CA3-CA1 LTP and many measures of synaptic plasticity including Glu-evoked spine enlargement, GluA1 receptor insertion, and CaMKII activation. Notably, loss of LTP did not appear to be due to alterations in basal synaptic transmission, as NMDA/AMPA current ratios, excitatory/inhibitory current ratios, paired-pulse facilitation, and input-output relationship of synaptic responses were all unchanged. Thus, the impairment of LTP appears to be due to block of a specific step in the signaling underlying synaptic plasticity. Further, analysis of cognitive functions showed that Orai1 KO mice show marked deficits in several hippocampal-dependent learning and memory behaviors, including working and associative memories (Figures 6 and 7), while sparing general sensorimotor functions, consistent with the lack of effects on basal synaptic transmission. Moreover, results in Orai1^*fl/fl CaMKIIa-Cre*^ mice show that deletion of Orai1 in excitatory neurons alone is sufficient to account for this learning and memory phenotype. We postulate that the deficits in synaptic plasticity and LTP at the CA3-CA1 synapse described here may extend to additional neural circuits and brain regions beyond the hippocampus, which could explain the striking deficits in associative learning. Together, these results establish a critical role for Orai1 channels in regulating biochemical processes involved in learning and memory in excitatory circuits.

How does Orai1 regulate synaptic plasticity? We find that Ca^2+^ elevations stimulated by Glu uncaging in mushroom-shaped dendritic spines are markedly smaller in Orai1 KO neurons (Figure 1), indicating that Orai1 plays a critical role in amplifying NMDAR-triggered dendritic Ca^2+^ elevations that are linked to synaptic plasticity. Interestingly, assessments of Ca^2+^ influx using Orai1-GCaMP6f indicated that Orai1 channels switch on rapidly (latency of 100–200 ms) in response to Glu uncaging and turn off quickly over 1–2 s following termination of the Glu-uncaging pulse. Moreover, changes in Orai1-mediated Ca^2+^ signals are tightly correlated with decreases in [Ca^2+^]_ER_ following synaptic stimulation (Figure 3), indicating that the kinetics of Orai1 channel activation are closely linked to the filling state of the ER. Notably, these kinetics of Orai1 activation are considerably faster than what would be expected from traditional models of SOCE activation in non-excitable cells, where activation rate is limited by the slow aggregation and accumulation of STIM1 and formation of Orai1-STIM1 punctae at the ER-plasma membrane junctions (Wu et al., 2006). Rather, the faster kinetics of Orai1 channel activation in dendritic spines are reminiscent of skeletal muscle, where recent reports indicate that SOCE activation and deactivation occur over timescales of milliseconds (Michelucci et al., 2018; Wei-Lapierre et al., 2013). In the skeletal muscle, rapid Orai1 channel activation is thought to arise from prepositioning of Orai1 channels in close apposition to STIM1 at the triad junctions even prior to store depletion, thereby eliminating the necessity of STIM1 to migrate to ER-PM junctions and trap Orai1 channels in the overlying plasma membrane (Michelucci et al., 2018; Wei-Lapierre et al., 2013). Our results raise the possibility that in an analogous manner, Orai1 channels may be prepositioned close to their ligand (perhaps STIM2; Sun et al., 2014) such that they are able to respond rapidly to store depletion stimuli to evoke Ca^2+^ influx and directly regulate spine [Ca^2+^]_i_.

On the basis of the findings presented here, we conclude that stimulation of synaptic GluRs on dendritic spines triggers [Ca^2+^]_i_ rises that are critically dependent on the opening of Orai1 channels (Figure S6). In line with previous proposals (Fitzjohn and Collingridge, 2002; Rose and Konnerth, 2001), opening of NMDARs by AMPAR-mediated depolarization of the postsynaptic membrane potential provides an initial trigger for Ca^2+^ entry. As shown by the measurements of Glu-uncaging-evoked decreases in [Ca^2+^]_ER_ (Figure 3), this in turn evokes rapid depletion of ER Ca^2+^ stores to stimulate Orai1 channel activation, presumably through the ER Ca^2+^ sensors, STIM1 and/or STIM2. In this scenario, although opening of NMDARs triggers the spine Ca^2+^ rise, Orai1 channels are required for amplification of the initial trigger Ca^2+^ signal to drive activation of essential Ca^2+^-dependent signaling pathways involved in LTP. More studies are required to understand the molecular mechanisms and functional architecture of this Ca^2+^ amplification process, including the mechanism(s) coupling ER Ca^2+^ stores to glutamate receptors and the molecular identity of the proximal gating signal that gates Orai1, but the results presented here provide a basis for testing these and other questions. Importantly, from a functional standpoint, the finding that Orai1 directly regulates dendritic spine Ca^2+^ store content and LTP is likely to have broad implications both for the basic mechanisms of synaptic plasticity as well as its potential contributions to the plasticity decline commonly seen in neurodegenerative diseases.

## STAR★METHODS

### RESOURCE AVAILABILITY

#### Lead Contact

Requests for resources and reagents should be directed and fulfilled by the Lead Contact, Dr. Murali Prakriya (m-prakriya@northwestern.edu).

#### Materials Availability

All unique/stable reagents generated in the study are available from the Lead Contact with a completed Materials Transfer Agreement.

#### Data and Code Availability

This study did not generate any unique data or codesets.

### EXPERIMENTAL MODEL AND SUBJECT DETAILS

#### Transgenic Mice

C57BL/6 mice were cared for in accordance with institutional guidelines and the Guide for the Care and Use of Laboratory Animals. Animals were group-housed in a sterile ventilated facility, under standard housing conditions (12/12 h light/dark cycle with lights on at 7 am, temperature 20-22°C with *ad libitum* access to water and food) and maintained with in-house breeding colonies. Male and female mice were used in approximately equal numbers. All research protocols were approved by the Northwestern University Institutional Animal Care and Use Committee. Neuronal cultures used neonatal (P0-P1) mice, slice electrophysiological studies used juvenile (P27-P38) mice, and behavioral studies used adult mice 9 to 15 weeks of age at the start of behavioral testing.

Tissue-specific deletion of Orai1 in the brain was accomplished as previously described (Hori et al., 2020; Somasundaram et al., 2014; Toth et al., 2019). Briefly, *Orai1*^*fl/fl*^ mice (provided by Amgen Inc.) and *Orai1*^*fl/+*^ were crossed with *nestin-Cre* mice (003771 from The Jackson Laboratory) to generate *Orai1*^*fl/fl Nes-Cre*^ (brain-specific KO) and *Orai1*^*fl/+ Nes-Cre*^ (brain-specific heterozygote). In addition to conditional deletion of Orai1 in the nervous system, the *Orai1*^*fl/fl*^ x *nestin-Cre* cross also produced germline transmission in some instances, resulting in *Orai1*^*fl/−*^ and *Orai1*^*fl/− Nes-Cre*^ genotypes, which were used in some cases for Ca^2+^ imaging experiments of Orai1 heterozygous and KO cultures, as indicated in the figure legends. To delete Orai1 selectively in excitatory (glutamatergic) neurons of the forebrain, Orai1^fl/fl^ mice were crossed with *Camk2a-Cre mice* (005359 from the Jackson Laboratory) (Tsien, 1998; Tsien et al., 1996) to yield Orai1^*fl/fl CaMKIIa-Cre*^ mice. Deletion in inhibitory interneurons was achieved by crossing Orai1^fl/fl^ mice with *Gad2-Cre mice* (010802 from the Jackson Laboratory) (Taniguchi et al., 2011) as previously described (Hori et al., 2020).

#### Cell culture

Primary hippocampal neurons were isolated from neonatal (P0-P1) mice by standard techniques (Toth et al., 2019) with minor modifications. Briefly, hippocampi were dissected and meninges removed under a dissection microscope in 4°C dissection medium (10 mM HEPES in HBSS). The tissue was minced and trypsinized with gentle mechanical agitation (0.25% trypsin; Invitrogen) for 10 min in a 37°C water bath in neuronal media consisting of Neurobasal supplemented with 2% B-27, 2 mM L-glutamine, and 1% penicillin-streptomycin. Dissociated neurons were seeded on Poly-D-coated coverslips (~20,000 cells/coverslip) and fed by Neuronal culture medium (Neurobasal medium GIBCO #21103049) containing B27, 1% L-Glutamine, and 1% Penicillin/Streptomycin. Prior to plating, the glass coverslips were cleaned using base piranha solution etching, and UV-sterilized for 30 minutes before culture. The culture medium was changed every fourth day and 100μM D-APV was supplemented starting at DIV 4 to minimize glutamate toxicity. Cultures were maintained up to 4 weeks and transfected with construct of interest at 21 DIV using Lipofectamine 2000 (Invitrogen # 11668027).

### METHOD DETAILS

#### Plasmids and transfection

Neurons were transfected using Lipofectamine 2000 (Invitrogen) according to the manufacturer’s instructions. The E106A Orai1-YFP plasmid has been described previously (Navarro-Borelly et al., 2008). jGCaMP7f (plasmid # 104483), G-CEPIA1er (plasmid #58215), red shifted R-CEPIA1er (plasmid # 58216), and Orai1-GCaMP6f (plasmid # 73564) were obtained from AddGene (https://www.addgene.org). mCherry-Orai1 was kind gift of Dr. Richard Lewis (Stanford University). Recycling of AMPAR was studied using SEP-GluA1 (GFP tagged) sensor, which was obtained from Dr. Antonio Sanz-Clemente (Northwestern University). CaMKII-α activity was monitored using the Camui sensor (GFP/RFP FRET), a kind gift from Dr. Yasunori Hayashi (Kyoto University). Orai1 mutants were generated by the QuikChange Mutagenesis kit (Agilent Technologies), and the mutations were confirmed by DNA sequencing. Experiments were performed 24-48 hours after transfection.

#### Microscopy

##### Ca^2+^ imaging using confocal microscopy

Confocal imaging was performed on a Nikon A1R upright confocal microscope using a 25X Nikon CFI APO LWD objective. Ca^2+^ signals in dendritic spines were monitored using the recently developed high-performance indicator, jGCaMP7f (Dana et al., 2019). Cells were co-transfected with mCherry to facilitate identification of dendrites and dendritic spines. Ca^2+^ measurements in single dendritic spines were determined from the intensity of GFP in single mushroom-shaped spines with a with a spine head/neck ratio >1.1 and spine diameter > 1 micron (spine area > 0.78μm^2^)(Lai and Ip, 2013; Nimchinsky et al., 2002; von Bohlen Und Halbach, 2009) (determined from the mCherry fluorescence). jGCaMP7f fluorescence was monitored on the resonant scanner of the A1R confocal scope at a scanning rate of 15 Hz. Cells were excited with a 488 nm laser line for jGCaMP7f measurements with a 525/50 nm emission filter. mCherry was excited at 561 nm and images collected using an 595/50 nm emission filter set. Images were taken at 1.1AU pinhole size using GaAsP detectors. Image capture and analysis was done using NIS-Elements software.

ER Ca^2+^ measurements were performed using either G-CEPIA1er (Addgene plasmid #58215) or the red shifted R-CEPIA1er (plasmid # 58216) as indicated in the figures. Orai1 channel activity was examined using Orai1-GCaMP6f (Addgene plasmid # 73564) (Dynes et al., 2016). mCherry-Orai1 and STIM1-mCherry were a gift from Dr. Lewis laboratory (Stanford University). All mutants of Orai1 were generated by the QuikChange Mutagenesis kit (Agilent Technologies), and the mutations were confirmed by DNA sequencing. Ca^2+^ measurements in single dendritic spines were determined from the intensity of GFP chosen by the described criterion described in the text using the resonant scanner of the A1R confocal scope with a scanning rate of 15 Hz. Simultaneous Cytosolic and ER Ca^2+^ measurements were done by sequentially imaging GFP (GCaMP7f) and RFP (R-CEPIA1er) at a scanning rate of 15Hz.

##### Camui and SEP-GluA1 imaging

Activation of CaMKII-α in dendritic spines was measured using the Camui sensor (GFP/RFP FRET) (Takao et al., 2005). Binding of Ca^2+^/CaM to Camui “opens” up the closed configuration of this CaMKII sensor, resulting in a decrease in FRET efficiency (Erickson et al., 2011; Takao et al., 2005). Camui was excited using the 488 nm excitation laser line and both the GFP and RFP emission were simultaneously monitored every 20 s. Recycling of AMPAR was examined using SEP-GluA1 sensor (GFP). SEP-GluA1 was excited using the GFP (488 nm) laser line and GFP and RFP emissions were simultaneously imaged every 20 s.

##### Glutamate uncaging

Alignment of laser stimulation point, duration of stimulation, and laser power to uncage compounds were optimized by a trial and error by uncaging CMNB-caged fluorescein. The galvanometer of the microscope was focused to a single pixel (~0.12μm/pixel), and 405nM laser was used to uncage CMNB-caged Fluorescein while the resonant scanner was used to image GFP fluorescence in order to probe the efficacy of photoconversion by a single pixel stimulation. Laser power for a 4ms uncaging pulse was adjusted to minimize the spread of the uncaged fluorescence to a 1 μm^2^ spot in each uncaging pulse (the standard laser power used was 0.16 mW). Control experiments with caged fluorescein indicated that a 4 ms uncaging pulse yielded a lateral spread of 2 μm Figures S2A–S2C). MNI-Caged-L-glutamate (Tocris#1490) was dissolved to a final concentration of 1mM in the external bath solution (Ringer’s solution) containing 1μM TTX. Single dendritic spines, located by the mCherry fluorescence (~1 μm in diameter), located 100-150 μm away from the soma on the secondary apical dendrites (typical thickness ~0.5 μm) were stimulated by uncaging MNI-glutamate. Glutamate was uncaged by 405 nM/20 mW laser at a single pixel positioned ~0.5 μm from the spine head in the direction away from the parent dendrite. Spines were typically stimulated for 4 ms using power values determined from uncaging CMNB-Fluorescein (laser power ~0.16 mW). This approach permitted stimulation of individual spines with high specificity. For experiments involving Orai1-GCaMP6f, we found that the fluorescence intensity of Orai1-GCaMP6f was significantly dimmer than that observed with *soluble* GCaMP6f or jGCaMP7f, suggesting that the expression of the GCaMP6f indicator is lower when it is tethered to the channel than when expressed by itself as a soluble protein (average GCaMP6f fluorescence (in A.U) in unstimulated cells = 104 ± 7; jGCaMP7f = 68 ± 7; and Orai1-GCaMP6f = 27 ± 2). Hence, we used stronger stimulation durations (64 ms uncaging) to detect fluorescence changes in the Orai1 KO cells expressing Orai1-GCaMP6f.

##### Widefield Ca^2+^ imaging

Primary hippocampal neurons grown on poly D-lysine coated glass-bottom dishes were loaded with fura-2 by incubating cells in 2μM Fura-2–AM (Invitrogen) in complete neurobasal medium for 30 min at 37°C. All experiments were performed at room temperature. Single cell [Ca^2+^]_i_ measurements were performed as described previously (Toth et al., 2019). Image acquisition and analysis were performed using Slidebook (Denver, CO). Dishes were mounted on the stage of an Olympus IX71 inverted microscope and images were acquired every 6 s at excitation wavelengths of 340 and 380 nm, and an emission wavelength of 510 nm. For data analysis, regions of interest were drawn around single cells, background was subtracted, and F340/F380 ratios were calculated for each time point. [Ca^2+^]_i_ was estimated from F340/F380 ratio using the standard equation: [Ca^2+^]_i_ = βK_d_ (R-R_min_)/(R_max_-R) where R is the F340 /F380 fluorescence ratio and values of R_min_ and R_max_ were determined from an *in vitro* calibration of Fura-2 penta-potassium salt. β was determined from the F_min_/F_max_ ratio at 380 nm and K_d_ is the apparent dissociation constant of Fura-2 binding to Ca^2+^ (135 nM). The values of these parameters were: R_min_ = 0.21, R_max_ = 5.056, β = 13.2. For each cell, the rate of SOCE (Δ[Ca^2+^]_i_/Δt) was calculated from the slope of a line fitted to three points (18 s) following the re-addition of 2 mM Ca^2+^ Ringer’s solution.

##### Brain slice preparation

Acute hippocampal slices were prepared from juvenile (P27-P38) mice of either gender for electrophysiology, in accordance with Institutional Animal Care and Use Committee-approved protocols. Mice were transcardially perfused with ice-cold sucrose-rich slicing artificial cerebral spinal fluid (aCSF) containing 85 mM NaCl, 2.5 mM KCl, 1.25 mM NaH_2_PO_4_, 25 mM NaHCO_3_, 75 mM sucrose, 25 mM glucose, 10 μM D-APV, 100 μM kynurenate, 0.5 mM Na L-ascorbate, 0.5 mM CaCl_2_, and 4 mM MgCl_2_, and oxygenated and equilibrated with 95% O_2_/5% CO_2_. Following perfusion, mice were decapitated. Animals were deeply anesthetized with isoflurane before decapitations and removal of the brains. Horizontal slices 350 μm were cut using a tissue slicer (Compresstome model VF-200-0Z, Precisionary Instruments) a Leica VT1200S vibratome (Leica Biosystems, Buffalo Grove, IL) in ice cold sucrose aCSF. For GCamP6f imaging, slices were cut in a solution containing (in mM): 110 choline chloride, 2.5 KCl, 25 NaHCO_3_, 1.25 NaH_2_PO_4_, 25 D-glucose, 11.6 ascorbic acid, 3.1 pyruvic acid, 7 MgCl_2_, and 0.5 CaCl_2_, saturated with 95% O_2_ and 5% CO_2_. Slices were then quickly transferred into a recovery chamber with aCSF solution maintained at 30°C and containing (in mM): 125 NaCl, 2.4 KCl, 1.2 Na_2_PO_4_, 25 NaHCO_3_, 25 glucose, 1 CaCl_2_ and 2 MgCl_2_ saturated with 95% O_2_ and 5% CO_2_. After 30 minutes in the recovery chamber, slices were transferred into a storage chamber with aCSF containing 2 mM CaCl_2_, where they were stored for 0.5-6 hours until they were used for electrophysiology.

##### Slice Electrophysiology

Field EPSPs (fEPSPs) were evoked by stimulating Schaffer collaterals with a monopolar glass electrode filled with aCSF at a basal stimulation frequency of 0.05 Hz. Glass electrodes were pulled from borosilicate glass to resistances of 1–3 Ω. fEPSPs were recorded in the CA1 apical dendrite layer using a glass pipette (2–5 Ω) filed with aCSF recording solution. Stimulus intensity was adjusted to induce a fEPSP amplitude ~40% of the maximal fEPSP. LTP was induced by 100 Hz high frequency stimulation (HFS) for 1 s. Data were collected at 20 kHz and filtered at 1 kHz. Data were excluded if the averaged fEPSP slope of initial 5 min and last 5 min in baseline differed by more than 5%. The amount of LTP was determined by normalizing the averaged fEPSP slope during the last 10 min of each experiment to the 20 min of basal control.

For voltage clamp and current clamp recordings, recording electrodes were pulled borosilicate glass with tip resistance of 4-7 MΩ. Data were collected at a sampling rate of 20 kHz and filtered at 5 kHz. In voltage clamp recordings, recording pipettes were filled with intracellular solution containing 95 mM CsF, 25 mM CsCl, 10 mM HEPES, 10 mM EGTA, 2 mM NaCl, 2 mM Mg-ATP, 10 mM QX-314, 5 mM TEA-Cl, and 5 mM 4-AP, and adjusted to pH 7.3 with CsOH. After whole-cell break-in, CA1 neurons were maintained in aCSF containing GABA_A_ receptor antagonists bicuculline (10 mM) and picrotoxin (50 μM) for NMDA/AMPA recordings, and bicuculline (10 μM), picrotoxin (50 μM) and NMDA receptor antagonist D-APV (50 μM) for paired pulse recordings. In sEPSC and sIPSC recordings, cesium methanesulfonate internal solution containing 100 mM Cesium methanesulfonate, 8 mM CsCl, 0.5 mM CaCl_2_, 10 mM HEPES, 5 mM EGTA, 5 mM Na_2_-phosphocreatine, 2 mM Mg-ATP, 0.5 mM 2Na-GTP, 5 mM QX-314, and adjusted to pH 7.2 with CsOH was loaded into recording electrodes. sEPSC and sIPSCs were automatically detected with the Minianalysis (Synaptosoft, Decatur, GA, USA) using the threshold of ‘sEPSC or sIPSC amplitude’, set at five times the root mean square noise of the baseline. Miniature EPSCs (mEPSCs) were recorded in the presence of 1 μM TTX.

In current clamp recordings, the internal solution contained 130 mM K-gluconate, 20 mM KCl, 1 mM K-HEPES, 0.2 mM EGTA, 0.3 mM Na-GTP, and 4 mM Mg-ATP, pH 7.3. After whole-cell break-in, CA1 neurons were maintained in aCSF containing bicuculline (10 μM), picrotoxin (50 μM), D-APV (50 μM) and the AMPA and kainate receptor antagonist NBQX (10 μM). To record input-output relationships, membrane potential was held initially at −70 mV before injecting −200 to 250 pA current in 25 pA increments. Liquid junction potential was calculated using pClamp 10 software (Molecular Devices, Sunnyvale, CA) and corrected for all recordings. Analyses were carried out with pClamp10 (Molecular Devices).

##### Immunohistochemistry

Orai1 expression was mapped by a monoclonal antibody targeted to the second extracellular loop of Orai1 (Abcam Cat# ab175040, 266.1) (Guzman et al., 2014). Anesthetized mice were perfused intracardially with saline and subsequently 4% PFA/1xPBS. Brains were extracted and post-fixed in 4% PFA for 24 hours at 4°C, followed by 48 hours in 30% sucrose. Free-floating sections were cut with a vibrotome at 40mm thickness. Antigen retrieval was performed using 10mM Na-Citrate in 1xPBSfor 10min at 55°C followed by autofluoresence quenching with 1%H_2_O_2_/1xPBS incubation for 30min at RT. Primary antibody was incubated in blocking solution containing 10% NGS, 0.25% triton, and 1%BSA. Secondary antibody was used at RT for 90min in blocking solution with 5% NGS in the presence of anti-mouse Alexa 488, (Invitrogen) at 1:500.

##### Golgi staining

7-8 weeks old mice were anesthetized by isoflurane inhalation and euthanized by a brief transcardial perfusion of PBS in accordance with Northwestern University Animal Care and Use Committee-approved protocols. Brains were then dissected, and impregnated for two weeks using FD Rapid GolgiStain kit (FD NeuroTechnologies, Columbia, MD) by following the manufacturer’s protocol. Horizontal slices (200μm) were cut using a Leica CM1900 cryostat (Leica Biosystems, Buffalo Grove, IL), stained, and mounted for confocal imaging. 30μm thick z sections were imaged for each brain using the A1R (Nikon, Inc.) confocal microscope. Images were first improved by 3D blind deconvolution using NIS-elements to minimize spherical aberrations. For each slice, 2000 μm of resolved secondary CA1 apical dendrites (located ~200μm from soma) were analyzed for the number of mature spines per each dendrite using Fiji. To compare spine sizes between WT and Orai1 KO neurons, spine area was quantified in 100 spines chosen from 5 secondary dendrites of 3 different slices per each genotype.

#### Animal Behavioral Analysis

All mice were allowed to habituate to the experiment room and the experimenter one week prior to the experiments. Male and female mice were 9 to 15 weeks of age at the start of testing. The experimenter was blinded to the genotype of each mouse during behavioral testing and analysis.

##### Y-maze

Mice were placed at the end of arm 3 in the Y-maze and allowed to enter any of the three arms for 8 min while activity was recorded by a video tracking system. The order of arm entries was analyzed manually for spontaneous alternation. A successful alternation was recorded for each set of three consecutive arm choices in which no repeated entries occurred. An unsuccessful alternation was recorded for each set of three consecutive arm choices in which a repeated entry occurred. An arm entry was counted if the entire body of the mouse (excluding tail) entered the arm. Each mouse was analyzed until it had completed 20 possible (successful and unsuccessful) alternations. The number of successful alternations was divided by the number of possible alternations for the outcome measure, % alternation. Mice were excluded if they did not enter enough arms to complete 20 possible alternations. Chance level of alternation is 50%.

##### Trace Fear conditioning

Contextual and tone-dependent fear conditioning in the Nes-Cre and littermate control mice was examined using a computer-controlled activity monitor and tone/shock generator (TSE Systems). Mice were placed in Context 1 (Plexiglas chamber with a wire shock grid floor) for 173 s, followed by a 30 s tone (10 kHz; 75 dB), followed by a 15 s pause (trace), followed by a foot shock (2 s; 0.7 mA; constant current). After 24 hours, the mice were placed in a novel context (Context 2) for 60 s, followed by exposure to the original tone for 30 s, followed by a 15 s trace. Freezing was measured every 10th second in the novel context, every 5th second during the tone, and every 3rd second during the trace. After 24 more hours, the mice were placed back in the original context 1 and contextual freezing was measured every 10th second during a 180 s exposure. The studies on the CaMKII-Cre and GAD2-Cre mice were done using the same protocol as described above but used a different apparatus due to lack of availability of the original facility used for the Nes-Cre studies. In this case, the mice trained and tested using Habitest modular system (Coulbourn, Holliston, MA) fear conditioning system with exactly the same protocol for contexts, tone, trace and foot shock. Freezing behavior was scored using FreezeFrame software (Acrimetrics, Wilmette, IL) by adjusting the motion index for each trial and minimum 1 s bouts of freezing.

##### Open field

Mice were placed in the center of an open arena (56 × 56cm), and ambulation activity for 5 min was recorded by LimeLight software (Actimetrics, Wilmette, IL). The open field was divided into a 5 × 5 grid of squares, with the outer 16 squares defining the ‘outer’ region and the inner 9 squares defining the ‘middle’ region. The software provided the total distance traveled, the percentage of time the mouse spent within each of the two regions, and the number of crossings from one region to the other.

##### Rotarod

Mice were tested on a Rotarod device (TSE Systems) over 5 days. For first three ‘training’ days, the mice were individually placed on the rod, which rotated at 12 rpm for 60 s. The time at which the mouse fell off the rod was recorded. If the mouse remained on the rod without falling for the duration of the trial, 60 s was recorded as the time for that trial. For the last two ‘test’ days, the mice were individually placed on the rod, which rotated beginning at 4 rpm and accelerated to 40 rpm over 5 min. The time at which the mouse fell off the rod was recorded. Four trials per day were conducted for each mouse, spaced approximately 1 hour apart.

### QUANTIFICATION AND STATISTICAL ANALYSIS

#### Image quantification and analysis

GCaMP, G-CEPIA1er, R-CEPIA1er, and SEP-GluA1 images were analyzed by selecting single spines as ROI. Each ROI was background subtracted and AFoF_0_ was calculated using NIS-Elements software. For the Camui studies, GFP and RFP intensities were first background subtracted, and the donor to acceptor GFP/RFP intensity ratios were determined and normalized to initial intensity ratio. To measure the delay between changes of cytosolic Ca^2+^ (GCaMP) in GFP channel, and ER (CEPIA) in mCherry channel, onset of the response of each channel was separately calculated. Onset of the response is detected as the instant that the respective signal changes more than 5-fold the standard deviation of basal ΔF/F_0_ (first 5 s of measurement before uncaging stimulus) in response to the uncaging pulse. To determine the dynamics of signals for ER or cytosolic Ca^2+^ levels, the 10%–90% rise time or decay time constant (τ) based on a single parameter exponential function, each measurement was fitted with the parameter of interest and then the calculated parameter was averaged to calculate the mean and SEM.

#### Statistics

Statistical significance was calculated using OriginPro and p values less than or equal to 0.05 were considered to be significant (*p < 0.05, **p < 0.01, ***p < 0.001). For datasets with two groups, statistical analysis was performed with two-tailed t test to compare between control and test conditions. For datasets with more than two groups, one-way ANOVA followed by Bonferroni post hoc mean comparison and Levene’stest of variance was used to compare groups. Ca^2+^ peaks were processed using Peak Analyzer function of OriginPro. A moving baseline with asymmetric least-squares smoothing was used, and peaks were determined from the local maxima that exceeded a threshold of 10% over the local baseline in a 2 s window following each stimulation. 10-90 rise time and exponential decay of Ca^2+^ and CEPIA signals were done using OriginPro. For exponential decay, a non-linear regression analysis was performed for each measurement to calculate the corresponding exponential decay (τ) parameter. N numbers are described in each figure legends and data is represented as mean ± SE.

## Supplementary Material

1

## Figures and Tables

**Table:** KEY RESOURCES TABLE

REAGENT or RESOURCE	SOURCE	IDENTIFIER
Antibodies		
Mouse anti-Orai1	Abcam	Cat# ab175040; RRID: AB_2877712
Goat anti-mouse Alexa 488	Thermofisher Scientific	Cat# A28175; RRID: AB_2610666
Bacterial and Virus Strains		
XL10-Gold Ultra-competent Cells	Agilent Technologies	Cat# 200315
MAX Efficiency DH5α Competent Cells	Invitrogen	Cat# 18258012
Chemicals, Peptides, and Recombinant Proteins		
Neurobasal Medium	Thermofisher Scientific	Cat# 21103049
B-27 Supplement	Thermofisher Scientific	Cat# 17504001
L-Glutamine	Thermofisher Scientific	Cat# 25030081
Lipofectamine 2000	Thermofisher Scientific	Cat# 11668019
Opti-MEM Reduced Serum Medium	Thermofisher Scientific	Cat# 31985088
DNase I	MilliporeSigma	Cat# 9003-98-9
Trypsin	Thermofisher Scientific	Cat# 15090046
Poly-D-Lysine	Thermofisher Scientific	Cat# A3890401
Fura-2, AM	Thermofisher Scientific	Cat# F1221
EGTA, AM	Thermofisher Scientific	Cat# E1219
MNI-caged-L-glutamate	R&D Systems	Cat# 1490
CMNB-Caged Fluorescein	Thermofisher Scientific	Cat# F7103
16% Formaldehyde	Thermofisher Scientific	Cat# 28906
Triton X-100	Thermofisher Scientific	Cat# 85111
Normal Goat Serum (NGS)	Thermofisher Scientific	Cat# 31873
ProLong Gold Antifade Mountant with DAPI	Thermofisher Scientific	Cat# P36935
Commercial Assays		
FD Rapid Golgi Stain Kit	FD Neuro Technologies	Cat# PK401A
QuikChange Site-Directed Mutagenesis Kit	Agilent Technologies	Cat# 200519
Experimental Models: Organisms/Strains		
Mouse: B6.Cg-Tg(Nes-cre)1Kln/J	The Jackson Laboratory	JAX: 003771
Mouse: B6.Cg-Tg(Camk2a-cre)T29-1Stl/J	The Jackson Laboratory	JAX: 005359
Mouse: STOCK Gad2tm2(cre)Zjh/J	The Jackson Laboratory	JAX: 010802
Mouse: Orai1 fl/fl	Amgen	N/A
Oligonucleotides		
Forward primer for WT Orai1 GATGAGCCTCAACGAGCACT	This paper	N/A
Reverse primer for WT Orai1 ATTGCCACCATGGCGAAGC	This paper	N/A
Forward primer for E106 Orai1 GTGGCAATGGTGGCGGTGCAGCTGGAC	This paper	N/A
Reverse primer for E106 Orai1 GTCCAGCTGCACCGCCACCATTGCCAC	This paper	N/A
Recombinant DNA		
Plasmid: mCherry	Dr. Scott Gradia	Addgene Plasmid #30125
Plasmid: jGCaMP7f	Dana et al., 2019	Addgene Plasmid #104483
Plasmid: GCaMP6f	Chen et al., 2013	Addgene Plasmid #40755
Plasmid: Orai1-GCaMP6f	Dynes et al., 2016	Addgene Plasmid #73564
Plasmid: Orai1 E106A-GCaMP6f	This paper	N/A
Plasmid: G-CEPIA1er	Suzuki et al., 2014	Addgene Plasmid #58215
Plasmid: R-CEPIA1er	Suzuki et al., 2014	Addgene Plasmid #58216
Plasmid: Camui	Gift of Dr. Yasunori Hayashi	Takao et al., 2005
Plasmid: SEP-GluA1	Gift of Dr. Antonio Sanz-Clemente	Makino and Malinow, 2009
Plasmid: mCherry-Orai1	Gift of Dr. Richard Lewis	Covington et al., 2010
Software and Algorithms		
Nikon Elements	Nikon	https://www.microscope.healthcare.mkon.com/products/software/nis-elements
ImageJ	NIH	https://imagej.nih.gov/ij/
OriginPro	OriginLab	https://www.originlab.com/index.aspx?go=Products/Origin

## Highlights

- Blockade of Orai1 activity impairs glutamate-evoked Ca^2+^ signals in dendritic spines of hippocampal neurons
- Orai1 channels promote synaptic plasticity including LTP in the hippocampus
- Deletion of Orai1 in excitatory neurons impairs working and associative memory
- Orai1 channels amplify dendritic spine Ca^2+^ signals to regulate cognitive functions

## References

[R1] Alford S, Frenguelli BG, Schofield JG, and Collingridge GL (1993). Characterization of Ca^2+^ signals induced in hippocampal CA1 neurones bythe synaptic activation of NMDA receptors. J. Physiol 469, 693–716. 8271224 10.1113/jphysiol.1993.sp019838 PMC1143895

[R2] Baba A, Yasui T, Fujisawa S, Yamada RX, Yamada MK, Nishiyama N, Matsuki N, and Ikegaya Y (2003). Activity-evoked capacitative Ca2+ entry: implications in synaptic plasticity. J. Neurosci 23, 7737–7741. 12944501 10.1523/JNEUROSCI.23-21-07737.2003 PMC6740588

[R3] Benson DL, Watkins FH, Steward O, and Banker G (1994). Characterization of GABAergic neurons in hippocampal cell cultures. J. Neurocytol 23, 279–295. 8089704 10.1007/BF01188497

[R4] Bloodgood BL, and Sabatini BL (2007). Ca(2+) signaling in dendritic spines. Curr. Opin. Neurobiol 17, 345–351. 17451936 10.1016/j.conb.2007.04.003

[R5] Bloodgood BL, Giessel AJ, and Sabatini BL (2009). Biphasic synaptic Ca influx arising from compartmentalized electrical signals in dendritic spines. PLoS Biol. 7, e1000190. 19753104 10.1371/journal.pbio.1000190 PMC2734993

[R6] Chen TW, Wardill TJ, Sun Y, Pulver SR, Renninger SL, Baohan A, Schreiter ER, Kerr RA, Orger MB, Jayaraman V, (2013). Ultrasensitive fluorescent proteins for imaging neuronal activity. Nature 499, 295–300. 23868258 10.1038/nature12354 PMC3777791

[R7] Covington ED, Wu MM, and Lewis RS (2010). Essential role for the CRAC activation domain in store-dependent oligomerization of STIM1. Mol. Biol. Cell 21, 1897–1907. 20375143 10.1091/mbc.E10-02-0145 PMC2877647

[R8] Dana H, Sun Y, Mohar B, Hulse BK, Kerlin AM, Hasseman JP, Tsegaye G, Tsang A, Wong A, Patel R, (2019). High-performance calcium sensors for imaging activity in neuronal populations and microcompartments. Nat. Methods 16, 649–657. 31209382 10.1038/s41592-019-0435-6

[R9] Deb BK, Chakraborty P, Gopurappilly R, and Hasan G (2020). SEPT7 regulates Ca^2+^ entry through Orai channels in human neural progenitor cells and neurons. Cell Calcium 90, 102252. 32682163 10.1016/j.ceca.2020.102252

[R10] Dou Y, Xia J, Gao R, Gao X, Munoz FM, Wei D, Tian Y, Barrett JE, Ajit S, Meucci O, (2018). Orai1 Plays a Crucial Role in Central Sensitization by Modulating Neuronal Excitability. J. Neurosci 38, 887–900. 29229703 10.1523/JNEUROSCI.3007-17.2017 PMC5783967

[R11] Dynes JL, Amcheslavsky A, and Cahalan MD (2016). Genetically targeted single-channel optical recording reveals multiple Orai1 gating states and oscillations in calcium influx. Proc. Natl. Acad. Sci. USA 113, 440–445. 26712003 10.1073/pnas.1523410113 PMC4720334

[R12] Dynes JL, Yeromin AV, and Cahalan MD (2020). Cell-wide mapping of Orai1 channel activity reveals functional heterogeneity in STIM1-Orai1 puncta. J. Gen. Physiol 152, e201812239. 32589186 10.1085/jgp.201812239 PMC7478869

[R13] Emptage N, Bliss TV, and Fine A (1999). Single synaptic events evoke NMDA receptor-mediated release of calcium from internal stores in hippocampal dendritic spines. Neuron 22, 115–124. 10027294 10.1016/s0896-6273(00)80683-2

[R14] Emptage NJ, Reid CA, and Fine A (2001). Calcium stores in hippocampal synaptic boutons mediate short-term plasticity, store-operated Ca^2+^ entry, and spontaneous transmitter release. Neuron 29, 197–208. 11182091 10.1016/s0896-6273(01)00190-8

[R15] Erickson JR, Patel R, Ferguson A, Bossuyt J, and Bers DM (2011). Fluorescence resonance energy transfer-based sensor Camui provides new insight into mechanisms of calcium/calmodulin-dependent protein kinase II activation in intact cardiomyocytes. Circ. Res 109, 729–738. 21835909 10.1161/CIRCRESAHA.111.247148 PMC3182829

[R16] Feske S (2019). CRAC channels and disease - From human CRAC channelopathies and animal models to novel drugs. Cell Calcium 80, 112–116. 31009822 10.1016/j.ceca.2019.03.004 PMC6545165

[R17] Feske S, Gwack Y, Prakriya M, Srikanth S, Puppel SH, Tanasa B, Hogan PG, Lewis RS, Daly M, and Rao A (2006). A mutation in Orai1 causes immune deficiency by abrogating CRAC channel function. Nature 441, 179–185. 16582901 10.1038/nature04702

[R18] Fitzjohn SM, and Collingridge GL (2002). Calcium stores and synaptic plasticity. Cell Calcium 32, 405–411. 12543099 10.1016/s0143416002001999

[R19] Gao C, Frausto SF, Guedea AL, Tronson NC, Jovasevic V, Leaderbrand K, Corcoran KA, Guzman YF, Swanson GT, and Radulovic J (2011). IQGAP1 regulates NR2A signaling, spine density, and cognitive processes. J. Neurosci 31, 8533–8542. 21653857 10.1523/JNEUROSCI.1300-11.2011 PMC3121195

[R20] Gao X, Xia J, Munoz FM, Manners MT, Pan R, Meucci O, Dai Y, and Hu H (2016). STIMs and Orai1 regulate cytokine production in spinal astrocytes. J. Neuroinflammation 13, 126. 27245842 10.1186/s12974-016-0594-7 PMC4886427

[R21] Gilmartin MR, Balderston NL, and Helmstetter FJ (2014). Prefrontal cortical regulation of fear learning. Trends Neurosci. 37, 455–464. 24929864 10.1016/j.tins.2014.05.004 PMC4119830

[R22] Guzman R, Valente EG, Pretorius J, Pacheco E, Qi M, Bennett BD, Fong DH, Lin FF, Bi V, and McBride HJ (2014). Expression of ORAII, a plasma membrane resident subunit of the CRAC channel, in rodent and non-rodent species. J. Histochem. Cytochem 62, 864–878. 25249026 10.1369/0022155414554926 PMC4244304

[R23] Heo DK, Lim HM, Nam JH, Lee MG, and Kim JY (2015). Regulation of phagocytosis and cytokine secretion by store-operated calcium entry in primary isolated murine microglia. Cell. Signal 27, 177–186. 25451082 10.1016/j.cellsig.2014.11.003

[R24] Herring BE, and Nicoll RA (2016). Long-Term Potentiation: From CaMKII to AMPA Receptor Trafficking. Annu. Rev. Physiol 78, 351–365. 26863325 10.1146/annurev-physiol-021014-071753

[R25] Higley MJ, and Sabatini BL (2012). Calcium signaling in dendritic spines. Cold Spring Harb. Perspect. Biol 4, a005686. 22338091 10.1101/cshperspect.a005686 PMC3312680

[R26] Hori K, Tsujikawa S, Novakovic MM, Yamashita M, and Prakriya M (2020). Regulation of chemoconvulsant-induced seizures by store-operated Orai1 channels. J. Physiol 10.1113/JP280119. PMC7896430 32851638

[R27] Jairaman A, and Prakriya M (2013). Molecular pharmacology of store-operated CRAC channels. Channels (Austin) 7, 402–414. 23807116 10.4161/chan.25292 PMC3913763

[R28] Jairaman A, Yamashita M, Schleimer RP, and Prakriya M (2015). Store-Operated Ca^2+^ Release-Activated Ca^2+^ Channels Regulate PAR2-Activated Ca^2+^ Signaling and Cytokine Production in Airway Epithelial Cells. J. Immunol 195, 2122–2133. 26238490 10.4049/jimmunol.1500396 PMC4621783

[R29] Kandel ER (2001). The molecular biology of memory storage: a dialogue between genes and synapses. Science 294, 1030–1038. 11691980 10.1126/science.1067020

[R30] Korkotian E, Oni-Biton E, and Segal M (2017). The role of the store-operated calcium entry channel Orai1 in cultured rat hippocampal synapse formation and plasticity. J. Physiol 595, 125–140. 27393042 10.1113/JP272645 PMC5199748

[R31] Kraeuter AK, Guest PC, and Sarnyai Z (2019). The Y-Maze for Assessment of Spatial Working and Reference Memory in Mice. Methods Mol. Biol 1916, 105–111. 30535688 10.1007/978-1-4939-8994-2_10

[R32] Kraft R (2015). STIM and ORAI proteins in the nervous system. Channels (Austin) 9, 245–252. 26218135 10.1080/19336950.2015.1071747 PMC4826113

[R33] Lai KO, and Ip NY (2013). Structural plasticity of dendritic spines: the underlying mechanisms and its dysregulation in brain disorders. Biochim. Biophys. Acta 1832, 2257–2263. 24012719 10.1016/j.bbadis.2013.08.012

[R34] Lee KF, Soares C, Thivierge JP, and Beique JC (2016). Correlated Synaptic Inputs Drive Dendritic Calcium Amplification and Cooperative Plasticity during Clustered Synapse Development. Neuron 89, 784–799. 26853305 10.1016/j.neuron.2016.01.012

[R35] Lein ES, Hawrylycz MJ, Ao N, Ayres M, Bensinger A, Bernard A, Boe AF, Boguski MS, Brockway KS, Byrnes EJ, (2007). Genome-wide atlas of gene expression in the adult mouse brain. Nature 445, 168–176. 17151600 10.1038/nature05453

[R36] Lisman J, Yasuda R, and Raghavachari S (2012). Mechanisms of CaMKII action in long-term potentiation. Nat. Rev. Neurosci 13, 169–182. 22334212 10.1038/nrn3192 PMC4050655

[R37] Lu W, Man H, Ju W, Trimble WS, MacDonald JF, and Wang YT (2001). Activation of synaptic NMDA receptors induces membrane insertion of new AMPA receptors and LTP in cultured hippocampal neurons. Neuron 29, 243–254. 11182095 10.1016/s0896-6273(01)00194-5

[R38] Luik RM, Wang B, Prakriya M, Wu MM, and Lewis RS (2008). Oligomerization of STIM1 couples ER calcium depletion to CRAC channel activetion. Nature 454, 538–542. 18596693 10.1038/nature07065 PMC2712442

[R39] Lynch MA (2004). Long-term potentiation and memory. Physiol. Rev 84, 87–136. 14715912 10.1152/physrev.00014.2003

[R40] Maciąg F, Majewski q., Boguszewski PM, Gupta RK, Wasilewska I, Wojtaś B, and Kuznicki J (2019). Behavioral and electrophysiological changes in female mice overexpressing ORAI1 in neurons. Biochim Biophys Acta Mol Cell Res 1866, 1137–1150. 30659848 10.1016/j.bbamcr.2019.01.007

[R41] Majewski Ł, Maciąg F, Boguszewski PM, Wasilewska I, Wiera G, Wójtowicz T, Mozrzymas J, and Kuznicki J (2017). Overexpression of STIM1 in neurons in mouse brain improves contextual learning and impairs long-term depression. Biochim Biophys Acta Mol Cell Res 1864, 1071–1087. 27913207 10.1016/j.bbamcr.2016.11.025

[R42] Majewski L, Wojtas B, Maciąg F, and Kuznicki J (2019). Changes in Calcium Homeostasis and Gene Expression Implicated in Epilepsy in Hippocampi of Mice Over expressing ORAI1. Int. J. Mol. Sci 20, 5539. 10.3390/ijms20225539 PMC6888010 31698854

[R43] Makino H, and Li B (2013). Monitoring synaptic plasticity by imaging AMPA receptor content and dynamics on dendritic spines. Methods Mol. Biol 1018, 269–275. 23681636 10.1007/978-1-62703-444-9_25

[R44] Makino H, and Malinow R (2009). AMPA receptor incorporation into synapses during LTP: the role of lateral movement and exocytosis. Neuron 64, 381–390. 19914186 10.1016/j.neuron.2009.08.035 PMC2999463

[R45] Malinow R, and Malenka RC (2002). AMPA receptor trafficking and synaptic plasticity. Annu. Rev. Neurosci 25, 103–126. 12052905 10.1146/annurev.neuro.25.112701.142758

[R46] Matsuzaki M, Honkura N, Ellis-Davies GC, and Kasai H (2004). Structural basis of long-term potentiation in single dendritic spines. Nature 429, 761–766. 15190253 10.1038/nature02617 PMC4158816

[R47] Michelucci A, García-Castarñeda M, Boncompagni S, and Dirksen RT (2018). Role of STIM1/ORAI1-mediated store-operated Ca^2+^ entry in skeletal muscle physiology and disease. Cell Calcium 76, 101–115. 30414508 10.1016/j.ceca.2018.10.004 PMC6290926

[R48] Moccia F, Zuccolo E, Soda T, Tanzi F, Guerra G, Mapelli L, Lodola F, and D’Angelo E (2015). Stim and Orai proteins in neuronal Ca(2+) signaling and excitability. Front. Cell. Neurosci 9, 153. 25964739 10.3389/fncel.2015.00153 PMC4408853

[R49] Navarro-Borelly L, Somasundaram A, Yamashita M, Ren D, Miller RJ, and Prakriya M (2008). STIM1-Orai1 interactions and Orai1 conformational changes revealed by live-cell FRET microscopy. J. Physiol 586, 5383–5401. 18832420 10.1113/jphysiol.2008.162503 PMC2655373

[R50] Nevian T, and Sakmann B (2004). Single spine Ca2+ signals evoked by coincident EPSPs and backpropagating action potentials in spiny stellate cells of layer 4 in the juvenile rat somatosensory barrel cortex. J. Neurosci 24, 1689–1699. 14973235 10.1523/JNEUROSCI.3332-03.2004 PMC6730461

[R51] Nimchinsky EA, Sabatini BL, and Svoboda K (2002). Structure and function of dendritic spines. Annu. Rev. Physiol 64, 313–353. 11826272 10.1146/annurev.physiol.64.081501.160008

[R52] Patterson MA, Szatmari EM, and Yasuda R (2010). AMPA receptors are exocytosed in stimulated spines and adjacent dendrites in a Ras-ERK-dependent manner during long-term potentiation. Proc. Natl. Acad. Sci. USA 107, 15951–15956. 20733080 10.1073/pnas.0913875107 PMC2936631

[R53] Prakriya M, and Lewis RS (2006). Regulation of CRAC channel activity by recruitment of silent channels to a high open-probability gating mode. J. Gen. Physiol 128, 373–386. 16940559 10.1085/jgp.200609588 PMC2151560

[R54] Prakriya M, and Lewis RS (2015). Store-Operated Calcium Channels. Physiol. Rev 95, 1383–1436. 26400989 10.1152/physrev.00020.2014 PMC4600950

[R55] Prakriya M, Feske S, Gwack Y, Srikanth S, Rao A, and Hogan PG (2006). Orai1 is an essential pore subunit of the CRAC channel. Nature 443, 230–233. 16921383 10.1038/nature05122

[R56] Raybuck JD, and Lattal KM (2014). Bridging the interval: theory and neurobiology of trace conditioning. Behav. Processes 101, 103–111. 24036411 10.1016/j.beproc.2013.08.016 PMC3943893

[R57] Rose CR, and Konnerth A (2001). Stores not just for storage intracellular calcium release and synaptic plasticity. Neuron 31, 519–522. 11545711 10.1016/s0896-6273(01)00402-0

[R58] Sabatini BL, Oertner TG, and Svoboda K (2002). The life cycle of Ca(2+) ions in dendritic spines. Neuron 33, 439–452. 11832230 10.1016/s0896-6273(02)00573-1

[R59] Sala C, and Segal M (2014). Dendritic spines: the locus of structural and functional plasticity. Physiol. Rev 94, 141–188. 24382885 10.1152/physrev.00012.2013

[R60] Sobczyk A, Scheuss V, and Svoboda K (2005). NMDA receptor subunit-dependent [Ca2+] signaling in individual hippocampal dendritic spines. J. Neurosci 25, 6037–6046. 15987933 10.1523/JNEUROSCI.1221-05.2005 PMC6725044

[R61] Somasundaram A, Shum AK, McBride HJ, Kessler JA, Feske S, Miller RJ, and Prakriya M (2014). Store-operated CRAC channels regulate gene expression and proliferation in neural progenitor cells. J. Neurosci 34, 9107–9123. 24990931 10.1523/JNEUROSCI.0263-14.2014 PMC4078087

[R62] Sun S, Zhang H, Liu J, Popugaeva E, Xu NJ, Feske S, White CL 3rd, and Bezprozvanny I (2014). Reduced synaptic STIM2 expression and impaired store-operated calcium entry cause destabilization of mature spines in mutant presenilin mice. Neuron 82, 79–93. 24698269 10.1016/j.neuron.2014.02.019 PMC4007018

[R63] Suzuki J, Kanemaru K, Ishii K, Ohkura M, Okubo Y, and lino M (2014). Imaging intraorganellar Ca2+ at subcellular resolution using CEPIA. Nat. Commun 5, 4153. 24923787 10.1038/ncomms5153 PMC4082642

[R64] Suzuki J, Kanemaru K, and lino M (2016). Genetically Encoded Fluorescent Indicators for Organellar Calcium Imaging. Biophys. J 111, 1119–1131. 27477268 10.1016/j.bpj.2016.04.054 PMC5034299

[R65] Takao K, Okamoto K, Nakagawa T, Neve RL, Nagai T, Miyawaki A, Hashikawa T, Kobayashi S, and Hayashi Y (2005). Visualization of synaptic Ca2+ /calmodulin-dependent protein kinase II activity in living neurons. J. Neurosci 25, 3107–3112. 15788767 10.1523/JNEUROSCI.0085-05.2005 PMC6725094

[R66] Takezawa R, Cheng H, Beck A, Ishikawa J, Launay P, Kubota H, Kinet JP, Fleig A, Yamada T, and Penner R (2006). A pyrazole derivative potently inhibits lymphocyte Ca2+ influx and cytokine production by facilitating transient receptor potential melastatin 4 channel activity. Mol. Pharmacol 69, 1413–1420. 16407466 10.1124/mol.105.021154

[R67] Taniguchi H, He M, Wu P, Kim S, Paik R, Sugino K, Kvitsiani D, Fu Y, Lu J, Lin Y, (2011). A resource of Cre driver lines for genetic targeting of GABAergic neurons in cerebral cortex. Neuron 71, 995–1013. 21943598 10.1016/j.neuron.2011.07.026 PMC3779648

[R68] Toresson H, and Grant SG (2005). Dynamic distribution of endoplasmic reticulum in hippocampal neuron dendritic spines. Eur. J. Neurosci 22, 1793–1798. 16197520 10.1111/j.1460-9568.2005.04342.x

[R69] Toth AB, Hori K, Novakovic MM, Bernstein NG, Lambot L, and Prakriya M (2019). CRAC channels regulate astrocyte Ca2+ signaling and gliotransmitter release to modulate hippocampal GABAergic transmission. Sci. Signal 12, eaaw5450. 31113852 10.1126/scisignal.aaw5450 PMC6837172

[R70] Tshuva RY, Korkotian E, and Segal M (2017). ORAI1-dependent synaptic plasticity in rat hippocampal neurons. Neurobiol. Learn. Mem 140, 1–10. 28189550 10.1016/j.nlm.2016.12.024

[R71] Tsien JZ (1998). Behavioral genetics: subregion- and cell type-restricted gene knockout in mouse brain. Pathol. Biol. (Paris) 46, 699–700. 9885822

[R72] Tsien JZ, Chen DF, Gerber D, Tom C, Mercer EH, Anderson DJ, Mayford M, Kandel ER, and Tonegawa S (1996). Subregion- and cell type-restricted gene knockout in mouse brain. Cell 87, 1317–1326. 8980237 10.1016/s0092-8674(00)81826-7

[R73] von Bohlen Und Halbach O (2009). Structure and function of dendritic spines within the hippocampus. Ann. Anat 191, 518–531. 19783417 10.1016/j.aanat.2009.08.006

[R74] Wegierski T, and Kuznicki J (2018). Neuronal calcium signaling via store-operated channels in health and disease. Cell Calcium 74, 102–111. 30015245 10.1016/j.ceca.2018.07.001

[R75] Wei-Lapierre L, Carrell EM, Boncompagni S, Protasi F, and Dirksen RT (2013). Orai1-dependent calcium entry promotes skeletal muscle growth and limits fatigue. Nat. Commun 4, 2805. 24241282 10.1038/ncomms3805 PMC3868675

[R76] Wu MM, Buchanan J, Luik RM, and Lewis RS (2006). Ca^2+^ store depletion causes STIM1 to accumulate in ER regions closely associated with the plasma membrane. J. Cell Biol 174, 803–813. 16966422 10.1083/jcb.200604014 PMC2064335

[R77] Wu YK, Fujishima K, and Kengaku M (2015). Differentiation of apical and basal dendrites in pyramidal cells and granule cells in dissociated hippocampal cultures. PLoS ONE 10, e0118482. 25705877 10.1371/journal.pone.0118482 PMC4338060

[R78] Zhang H, Wu L, Pchitskaya E, Zakharova O, Saito T, Saido T, and Bezprozvanny I (2015). Neuronal Store-Operated Calcium Entry and Mushroom Spine Loss in Amyloid Precursor Protein Knock-In Mouse Model of Alzheimer’s Disease. J. Neurosci 35, 13275–13286. 26424877 10.1523/JNEUROSCI.1034-15.2015 PMC4588605

[R79] Zucker RS, and Regehr WG (2002). Short-term synaptic plasticity. Annu. Rev. Physiol 64, 355–405. 11826273 10.1146/annurev.physiol.64.092501.114547

