# Orai1 channels are essential for amplification of glutamate-evoked Ca2+ signals in dendritic spines to regulate working and associative memory

**DOI:** 10.1016/j.celrep.2021.108911

**Published:** 2021-03-30

**Authors:** Mohammad Mehdi Maneshi, Anna B. Toth, Toshiyuki Ishii, Kotaro Hori, Shogo Tsujikawa, Andrew K. Shum, Nisha Shrestha, Megumi Yamashita, Richard J. Miller, Jelena Radulovic, Geoffrey T. Swanson, Murali Prakriya

In the originally published version of this article, Figure S1G showed an image of Orai1 expression from the Allen Brain Atlas website. Because of an error in the unsupervised search output of the Allen Brain Atlas site that tagged an unrelated protein for Orai1, we inadvertently showed an image of the unrelated protein. The panel has been updated with the correct image of Orai1 mRNA expression from the Allen Brain Atlas (verified by re-blasting with the template probe), and the corrected Figure S1 now appears here and with the revised supplemental information online. This change does not affect the results or conclusions of our study.

The authors regret this error.

## Supplementary Material

1

