# Supplementary material for: Orai1 Channels Are Essential for Amplification of Glutamate-Evoked Ca2+ Signals in Dendritic Spines to Regulate Working and Associative Memory
Source: Cell Rep. Author manuscript; Available in PMC 2021 Jan 25. (PMC7832685; doi:10.1016/j.celrep.2020.108464)
Supplement: 1 [file NIHMS1651255-supplement-1.pdf]

Cell Reports, Volume *33*

Supplemental Information

Orai1 Channels Are Essential for Amplification of Glutamate-Evoked Ca^{2+} Signals in Dendritic Spines to Regulate Working and Associative Memory

Mohammad Mehdi Maneshi, Anna B. Toth, Toshiyuki Ishii, Kotaro Hori, Shogo Tsujikawa, Andrew K. Shum, Nisha Shrestha, Megumi Yamashita, Richard J. Miller, Jelena Radulovic, Geoffrey T. Swanson, and Murali Prakriya

SUPPLEMENTARY MATERIAL

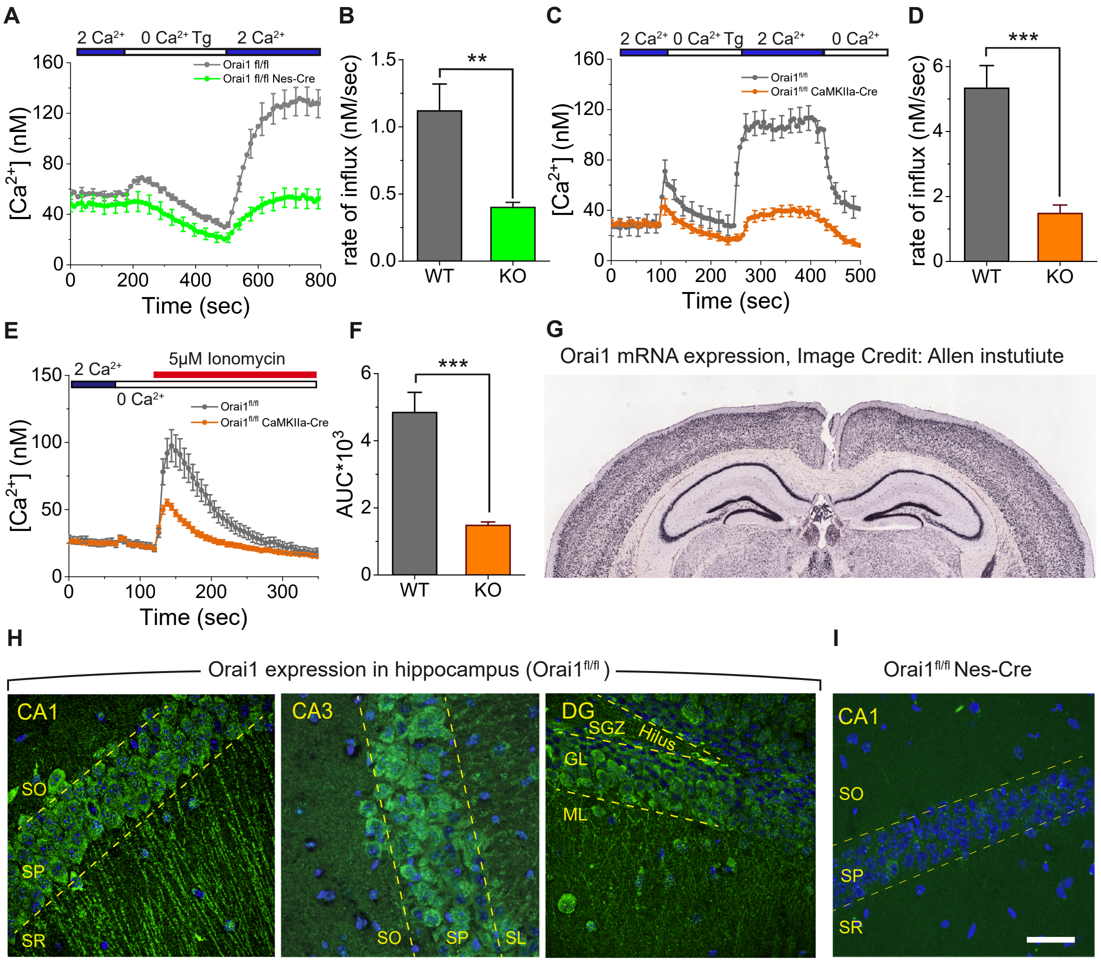

Supplementary Figure 1. Deletion of Orai1 abrogates SOCE in primary cultured hippocampal neurons. (Related to Figures 1 and 2). (A) SOCE in WT and Orai1 KO (Orai1$^{fl/fl\ Nes\text{-}Cre}$) mice. ER Ca^{2+} stores were depleted by administering thapsigargin (Tg ,1 μM) in a Ca^{2+}-free solution and SOCE was measured during readdition of extracellular Ca^{2+}. **(B)** Summary of the rate of Ca^{2+} influx following readdition of extracellular Ca^{2+} in WT and Orai1 KO neurons. (n=17 WT, 12 KO cells, p=0.00835). **(C,D)** SOCE is markedly attenuated in hippocampal neurons from Orai1$^{fl/fl\ CaMKIIa\text{-}Cre}$ mice. (n=49 WT, n=40 KO cells, p=0.00051). **(E)** Intracellular stores are reduced in Orai1 KO (Orai1$^{fl/fl\ CaMKIIa\text{-}Cre}$) neurons. Intracellular Ca^{2+} store content was assessed by discharging Ca^{2+} stores with 5 μM ionomycin in a Ca^{2+}-free Ringer's solution. **(F)** Quantification of the Ca^{2+} store content by integrating the area (over 180 s) under the curves in *E*. (n= 55 (WT), 52 (KO) cells, $p=1.71\times10^{-5}$). **(G)** Pattern of Orai1 mRNA expression in the mouse brain (Lein et al., 2015). Allen Brain Atlas API. Available from: brain-map.org/api/index.html. **(H)** Immunohistochemistry reveals significant expression of the Orai1 protein in the CA1, CA3, and dentate gyrus. SO: *Stratum Oriens*, SR: *Stratum Radiatum*, SP: *Stratum Pyramidale*, SGZ: subgranular zone, GL: granule cell layer; ML: molecular layer. **(I)** Orai1 staining is lost in littermate KO mice (Orai1$^{fl/fl\ Nes\text{-}Cre}$). Image shows the CA1 region. Scale bar, 50 μm.

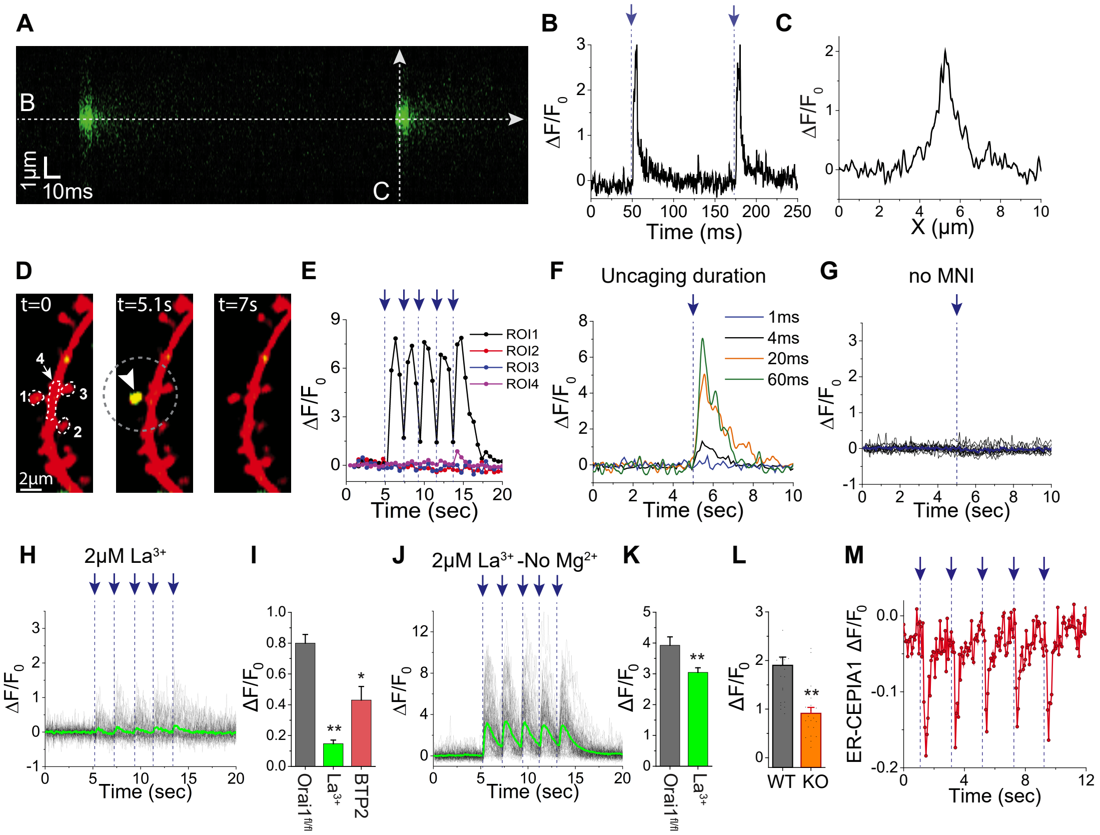

Supplementary Figure 2. Analysis of glutamate uncaging-evoked Ca^{2+} responses and related controls. (**Related to Figures 1 and 2**). **(A)** Line scans showing the temporal (*x*-axis) and lateral (*y*-axis) spread of fluorescein intensity following uncaging of caged fluorescein. **(B)** Kinetics of the change in fluorescein intensity at the spot (dotted line) shown in panel *A*. **(C)** Lateral spread of fluorescein intensity 4 ms following administration of the uncaging pulse. **(D)** A section of the secondary apical dendrite with several mushroom-shaped spines. The arrowhead in the middle panel denotes the spine stimulated by uncaging MNI-glutamate. ROIs denoting other nearby spines and the parent dendrite are also labelled. **(E)** Changes in jGCaMP7f fluorescence in the four ROIs from panel *D*. Except for the stimulated spine, the other spines and the parent dendrite do not show significant elevations in $[Ca^{2+}]_i$. **(F)** The magnitude of the jGCaMP7f fluorescence $\Delta F/F_0$ progressively increases with increasing stimulation duration (1-60 ms). **(G)** No change in jGCaMP7f fluorescence is seen when MNI-glutamate is omitted in the extracellular solution. **(H-I)** The magnitude of $[Ca^{2+}]_i$ change is strongly reduced by a low dose of La^{3+} (2 μM) and by the CRAC channel inhibitor, BTP2 (5 μM). The inhibitors were added to the extracellular solution in WT neurons. (n=8 WT, n=7 La^{3+}, n=6 BTP2 treated cells, p=0.0017, p=0.0047). **(J, K)** In the absence of extracellular Mg^{2+}, which should relieve NMDA receptor inhibition, the effect of 2 μM La^{3+} is greatly diminished. **(L)** Summary of the Ca^{2+} rises ($\Delta F/F_0$) in response to 64 ms duration uncaging pulses. When the uncaging duration is increased to 64 ms, the impairment of the Ca^{2+} signal in dendritic spines is smaller than when stimulated with a 4 ms pulse. (n=5 WT cells, 25 spines; n=6 Orai1-KO cells, 28 spines, p<0.0085). **(M)** CEPIAer fluorescence changes in response to a train of five 4 ms uncaging pulses.

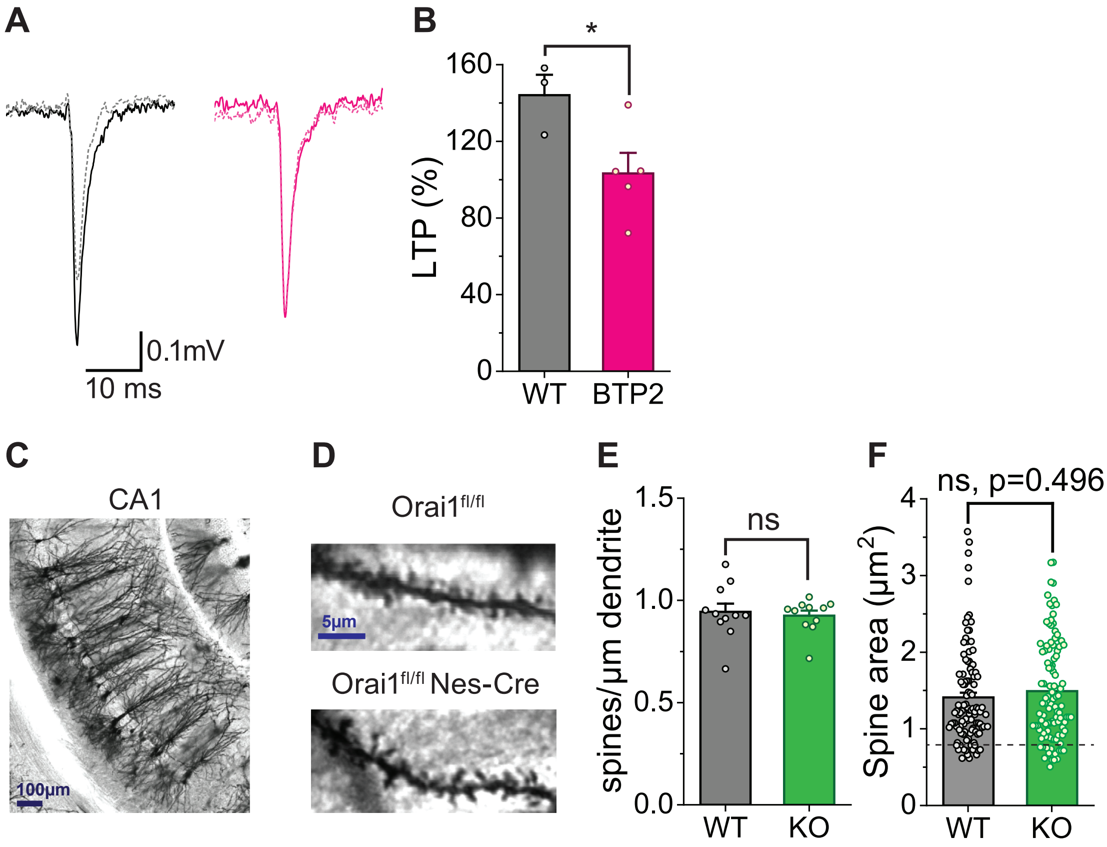

Supplementary Figure 3. Pharmacological inhibition of CRAC channels with BTP2 impairs LTP. (Related to Figure 5). (A) Representative traces of the fEPSPs in WT (Orai1$^{fl/fl}$) and BTP2-treated slices before and after tetanus (1x100 Hz). **(B)** Summary of the fEPSP slopes in control slices (WT, Orai1$^{fl/fl}$) and slices treated with 5µM BTP2. n=3 (WT slices), n=5 (BTP2-treated slices). p =0.0454. (**C**) CA1 pyramidal neurons visualized by Golgi-Cox rapid stain. (**D**) Representative examples of the secondary apical dendrites of WT (Orai1$^{fl/fl}$) and KO (Orai1$^{fl/fl\ Nes\text{-}Cre}$) mice in the CA1 region. (**E**) Summary of the number of mature spines per unit length (analyzed over 2000 µm of dendritic length) of secondary apical dendrites in the CA1 hippocampus. Mature spines were defined as spines with a head/neck ratio of >1.1 and spine head diameter of 1 µm (Lai and Ip, 2013; Nimchinsky et al., 2002; von Bohlen Und Halbach, 2009). **(F)** Summary of the cross-sectional area of spines located on secondary apical dendrites in CA1. (n=106 spines per genotype, p=0.496). The dashed line denotes the 0.78 µm^2 threshold area for mature spines.

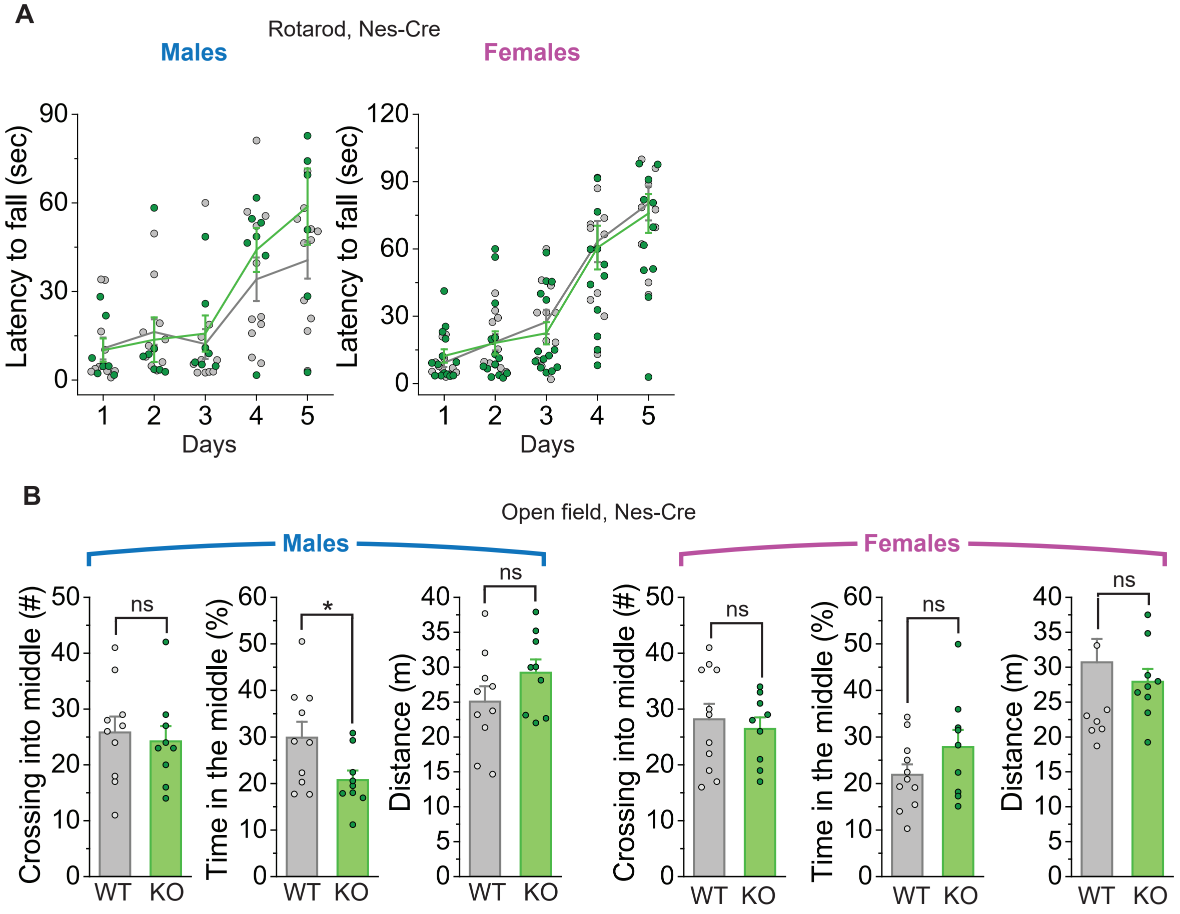

Supplementary Figure 4. Sensorimotor functions are not affected in the brain-specific Orai1 KO mice. (Related to Figure 6). (A) Rotarod analysis shows no differences between WT (Orai1$^{fl/fl}$) and KO (Orai1$^{fl/fl\ Nes\text{-}Cre}$) mice in the latency to fall over a 5-day period. **(B)** Likewise, no differences in Orai1 KO (Orai1$^{fl/fl\ Nes\text{-}Cre}$) mice were observed in the open-field test in the number of crossings in the middle of the test field, time spent in the middle of the field, or distance travelled.

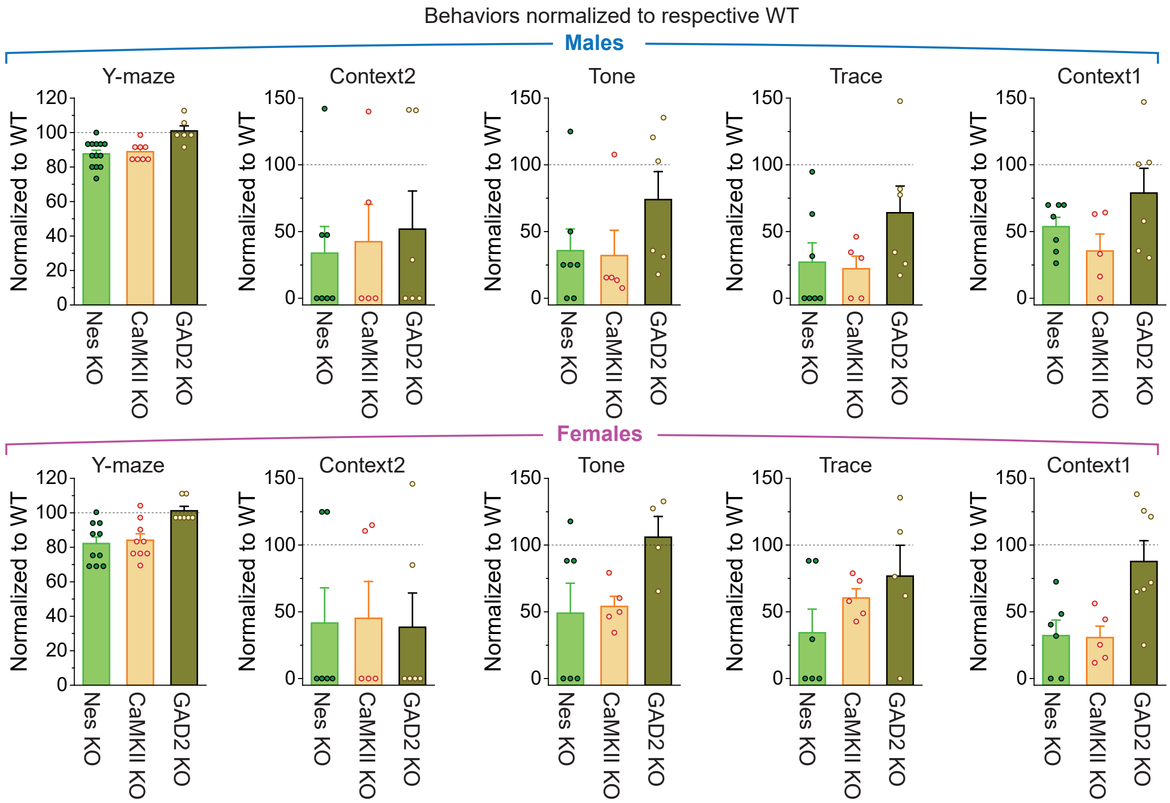

Supplementary Figure 5. Comparative analysis of learning and memory behaviors in the global and cell-specific Orai1 KO mice (Related to Figures 6 and 7). The data shown in Figure 7 were normalized to the corresponding WT values and directly compared across the three genotypes (Orai1$^{fl/fl\ Nes\text{-}Cre}$, Orai1$^{fl/fl\ CaMKIIa\text{-}Cre}$, and Orai1$^{fl/fl\ Gad2\text{-}Cre}$) to assess differences between the three types of Orai1 KO mice. Both in the Y-maze and in the fear-conditioning tests (context 1), the *Nes-Cre* and *CamKIIa-Cre* Orai1 KOs have significantly greater deficits compared to the Gad2-Cre Orai1 KOs, indicating that Orai1 function in *excitatory* neurons is critical for short-term and associative memory.

s

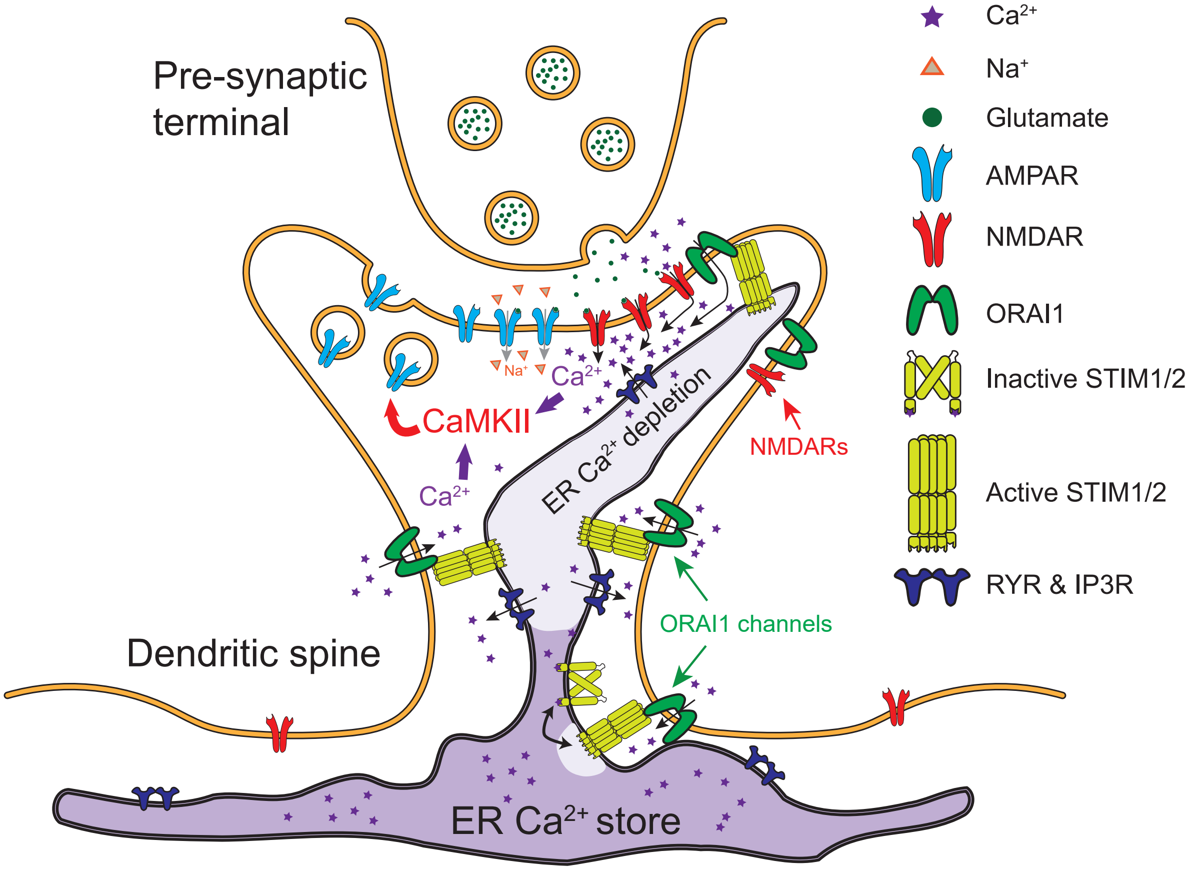

Supplementary Figure 6. A schematic of the proposed model for Orai1-mediated amplification of synaptically-evoked Ca^{2+} signals in dendritic spines. (Related to Figures 1, 5, 6, and 7). Stimulation of synaptic glutamate receptors evokes a trigger Ca^{2+} signal through NMDARs which, by itself, is insufficient to activate effector signaling pathways required for LTP. Instead, opening of NMDARs triggers CICR to deplete ER Ca^{2+} stores and activate Orai1 channels, likely via binding of the STIM proteins to Orai1. The ensuing Orai1-mediated Ca^{2+} entry strongly magnifies spine $[Ca^{2+}]$ elevations to stimulate activity-dependent synaptic plasticity pathways and enable learning and memory.
