# Supplementary material for: Orai1 channels are essential for amplification of glutamate-evoked Ca2+ signals in dendritic spines to regulate working and associative memory
Source: Cell Rep. Author manuscript; Available in PMC 2021 May 11. (PMC8112297; doi:10.1016/j.celrep.2021.108911)
Supplement: 1 [file NIHMS1689234-supplement-1.pdf]

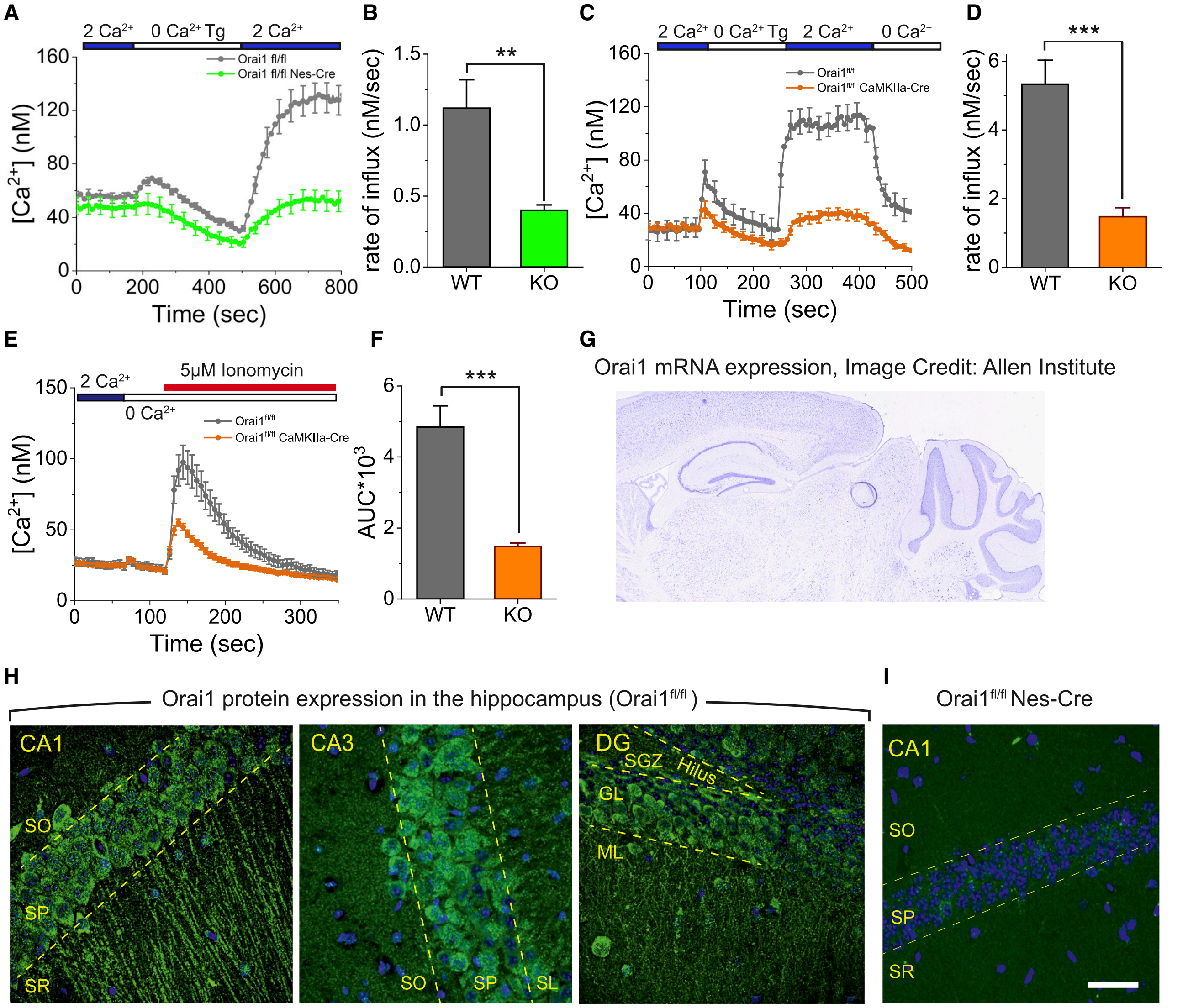

Figure S1. Deletion of Orai1 abrogates SOCE in primary cultured hippocampal neurons (related to Figures 1 and 2) (corrected)

CellPress
OPEN ACCESS

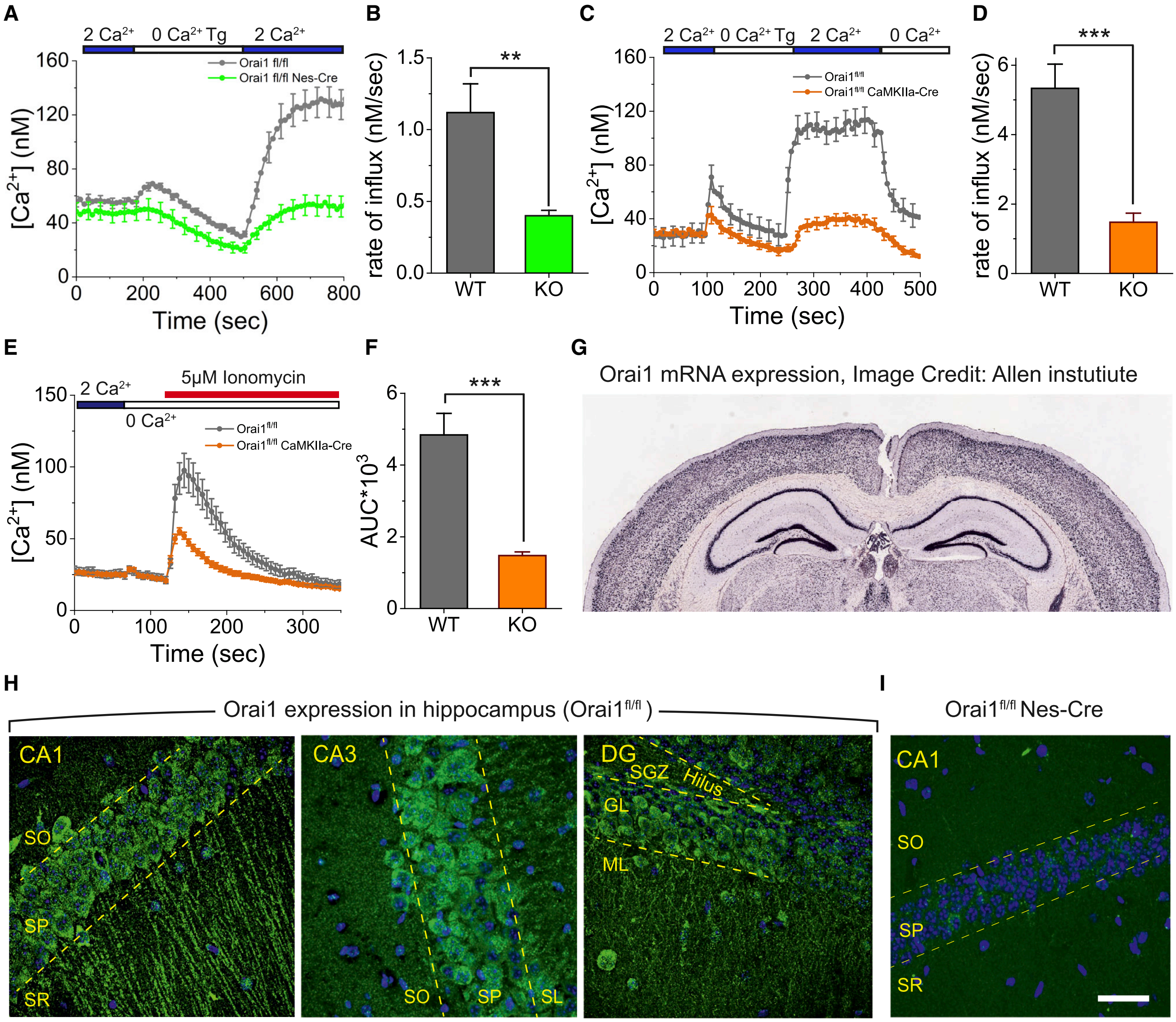

Figure S1. Deletion of Orai1 abrogates SOCE in primary cultured hippocampal neurons (related to Figures 1 and 2) (original)
